# Making Decisions under Ambiguity: Judgment Bias Tasks for Assessing Emotional State in Animals

**DOI:** 10.3389/fnbeh.2016.00119

**Published:** 2016-06-09

**Authors:** Sanne Roelofs, Hetty Boleij, Rebecca E. Nordquist, Franz Josef van der Staay

**Affiliations:** ^1^Department of Farm Animal Health, Faculty of Veterinary Medicine, Behavior and Welfare Group (Formerly Emotion and Cognition Group), Utrecht UniversityUtrecht, Netherlands; ^2^Department of Animals in Science and Society, Faculty of Veterinary Medicine, Division of Laboratory Animal Science, Utrecht UniversityUtrecht, Netherlands; ^3^Brain Center Rudolf Magnus, Utrecht UniversityUtrecht, Netherlands

**Keywords:** cognitive bias, emotion, trait, state, cognition, discrimination learning, go/no-go task, go/go task

## Abstract

Judgment bias tasks (JBTs) are considered as a family of promising tools in the assessment of emotional states of animals. JBTs provide a cognitive measure of optimism and/or pessimism by recording behavioral responses to ambiguous stimuli. For instance, a negative emotional state is expected to produce a negative or pessimistic judgment of an ambiguous stimulus, whereas a positive emotional state produces a positive or optimistic judgment of the same ambiguous stimulus. Measuring an animal’s emotional state or mood is relevant in both animal welfare research and biomedical research. This is reflected in the increasing use of JBTs in both research areas. We discuss the different implementations of JBTs with animals, with a focus on their potential as an accurate measure of emotional state. JBTs have been successfully applied to a very broad range of species, using many different types of testing equipment and experimental protocols. However, further validation of this test is deemed necessary. For example, the often extensive training period required for successful judgment bias testing remains a possible factor confounding results. Also, the issue of ambiguous stimuli losing their ambiguity with repeated testing requires additional attention. Possible improvements are suggested to further develop the JBTs in both animal welfare and biomedical research.

## Introduction

Within the framework of animal welfare studies as well as in biomedical studies, assessment of the emotional state of an animal can yield highly relevant information. The majority of studies on animal emotions (most of them using rodent species) address anxiety, which is assessed with classical tests such as the open field test (OF), the light-dark (LD) test and the plus-maze (PM) test (for a recent critique of these tests see Ennaceur, 2014). These tests measure the unconditioned response of an animal to an unfamiliar situation (the testing environment) that contains elements which the animal perceives as adverse/threatening (such as open space and/or high light intensities). They may be less suited for assessing emotion in non-rodent species such as pigs (see, e.g., Murphy et al., 2014). Instead of looking at the animal’s response to unconditioned stimuli, one may use cognitive tests to assess emotion in animals, such as judgment bias tasks (JBTs; Harding et al., 2004; Paul et al., 2005; Murphy et al., 2013), or (variants of) decision making tasks (Murphy et al., 2015). Affective reactions may provide useful feedback, both explicitly and implicitly, from emotional appraisal processes (Storbeck and Clore, 2007). According to Marchant-Forde (2015), the most influential recent studies measuring emotional state as an index of animal welfare are those assessing judgment (cognitive) bias. Bateson and Nettle (2015) consider JBTs as the “gold standard” for measuring the mood of animals. In the area of biomedical research, cognitive bias research is still in its infancy, although the number of studies using this type of task is growing. Besides its value for the purpose of welfare assessment, within the area of biomedical research, the affective state of an animal may be a confound for other behavioral tests and a source of uncontrolled variation (Bateson and Nettle, 2015). Knowledge of the animals’ emotional state may contribute to understanding test results.

### Emotion, Cognition and Judgment Bias

Emotions are adaptive processes that help individuals react adequately to internal or external stimuli, thereby avoiding harm and seeking valuable resources, while cognition can be described as information processing mechanisms. Emotions cannot be regarded separately from cognition. Emotional states affect cognitive processes and conversely cognitive processes are often the initiators of emotions (Lazarus, 1982; Dolcos, 2015). The interdependence of emotion and cognition is reflected in the definition by Kleinginna and Kleinginna, (1981 p. 355):

- “Emotion is a complex set of interactions among subjective and objective factors, mediated by neural–hormonal systems, which can
- (a) give rise to affective experiences such as feelings of arousal, pleasure/displeasure;
- (b) generate cognitive processes such as emotionally relevant perceptual effects, appraisals, labeling processes;
- (c) activate widespread physiological adjustments to the arousing conditions; and
- (d) lead to behavior that is often, but not always, expressive, goal-directed, and adaptive.”

The brain cannot be divided in cognitive and affective regions, since “affective” brain regions are also involved in cognition and brain regions that are viewed as cognitive are also involved in emotions. Cognition and emotion are integrated in the brain (Pessoa, 2008). Brain structures at the heart of the neural circuitry for emotion (e.g., the amygdala) impact cognitive processing from early attention allocation (Holland and Gallagher, 1999) through perceptual processing to memory (Phelps, 2006). Similarly, brain structures involved in the neural circuitry for cognition, such as dorsomedial and ventrolateral prefrontal cortex (DMPFC and VLPFC), have an intrinsic role in the experience of emotion (Barrett et al., 2007).

If one regards emotion as a result of an anticipated, experienced, or imagined outcome of an adaptationally relevant transaction between organism and environment, cognitive processes are always crucial in the elicitation of an emotion (Lazarus, 1982). Cognitive processes are closely linked to emotional states as they are, for example, necessary for the appraisal of environmental cues and for the “production” of emotions (Lazarus, 1982; Mathews and MacLeod, 1994). On the other hand, emotional states influence information processing in the brain, which helps individuals to react appropriately within a certain context (Mathews et al., 1997). Emotional influences on cognition are defined as cognitive biases, of which three types can be distinguished: attention biases, memory biases, and interpretation or judgment biases (see Paul et al., 2005). However, ascribing a reaction to a cue or stimulus to a *cognitive* bias implies that there is an unbiased, verifiable truth. It is, therefore, better to consider this phenomenon as result of “decision under ambiguity”, i.e., “judgment bias” instead of “cognitive bias”. Attention bias occurs in threatening situations as a result of an anxious emotional state and is characterized by an increased attention to negative and threatening cues (Mathews and MacLeod, 1994; Mogg and Bradley, 1998). Memory bias refers to the fact that events associated with positive or negative emotions are more readily remembered than neutral events, and includes memory storage, consolidation and retrieval processes (Cahill and McGaugh, 1996; Hamann et al., 1999). It is likely though, that the effects on memory are caused by high arousal and not by the valence of the emotion (Bradley et al., 1992). Judgment bias or interpretation bias (from now on referred to as judgment bias) refers to the influence of emotions on the interpretation of ambiguous information (Mathews et al., 1989; Eysenck et al., 1991; Richards and French, 1992).

There are numerous operational definitions of judgment bias. Combining definitions of Boleij et al. (2012) and Bateson and Nettle (2015),

*A judgment bias is a relative reaction to an ambiguous stimulus, expressing an “interpretation” of this stimulus and an “expectation” about the consequences of the reaction* (Boleij et al., 2012)*.* In JBTs* “(…) animals that respond to the ambiguous stimuli similarly to the positive stimulus are interpreted as displaying a high expectation of reward in the presence of ambiguous information, and hence an “optimistic” cognitive style indicative of a positive affective state. In contrast, animals that respond to the ambiguous stimuli similarly to the negative stimulus are interpreted as displaying a higher expectation of punishment or lower expectation of reward, and hence a more “pessimistic” cognitive style indicative of a more negative affective state”.*

*—(Bateson and Nettle, 2015, p. 3)*.

The processing of current information and the resulting behavioral choices are affected by optimism and pessimism (Dember et al., 1989). In JBTs, optimism is operationally defined “as a higher proportion of responses to an ambiguous cue as if it were the cue predicting the positive outcome, and pessimism as a higher proportion of responses to an ambiguous cue as if it were the cue predicting the negative outcome” (Douglas et al., 2012, p. 66). JBTs thus are believed to provide a cognitive measure of mood (Bateson et al., 2015; Mellor, 2015).

### Aim of this Review

Since its introduction as a test for use in animals just a decade ago (Harding et al., 2004), a considerable number of JBTs has been developed and applied in scientific studies, using a broad range of procedures and test equipment, in a large number of species (see “Supplementary Material, Table 1”, summarizing the publications about JBTs *with animals as subjects*). For continued (translational) research on animal emotions using these tasks, JBTs have to be validated and adapted to the abilities of each of these species (Anderson et al., 2012). Here, we review the different implementations of this task, and a number of questions to be solved, such as the cues and test arena’s used, measuring response latencies vs. categorizing responses as Go/No-go, and species-specific modifications. We discuss the potential of JBTs as a tool for measuring animals’ emotional state and to assess the effects of experimental manipulations of the emotional state. Outstanding questions for future research on measuring judgment bias in animals relevant for both animal welfare research and biomedical research are discussed.

## Discrimination Learning

**Figure 1:**
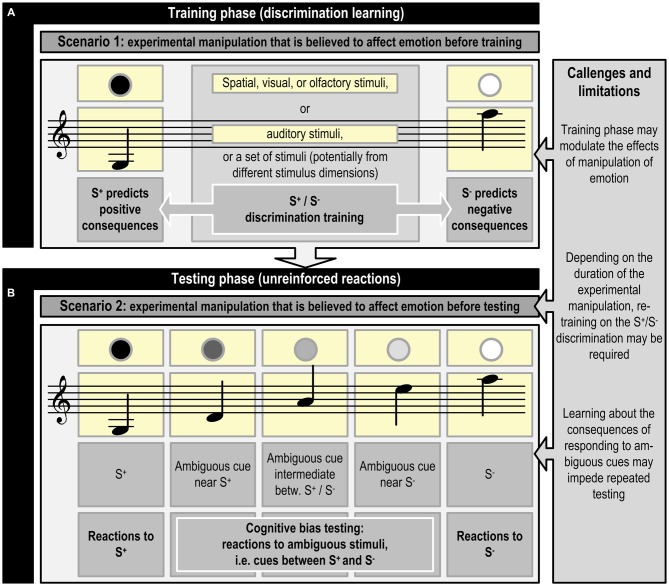
**Schematic representation of judgment bias training and testing using visual, olfactory, spatial, or auditory cues, or a combination of cues from different stimulus dimensions (inspired by Bateson et al., 2011; Mendl et al., 2011).** The experimental manipulation that is believed to affect emotion precedes the training phase (**A**; Scenario 1) or the testing phase (**B**; Scenario 2). Refreshment of the discrimination acquired during the training phase may be necessary, if the experimental manipulation preceding phase **(B)** lasts for a longer time period. An example of scenario 1 is studying the effects of growing up in different housing systems, whereas scenario 2 may be applied in a study assessing the effects of shorter lasting experimental manipulations, such as confinement, on emotion. Phase **(B)** may be repeated multiple times (e.g., Douglas et al., 2012) to test the effects of different experimental manipulations in the same animal. Specific challenges and limitations may be connected to the different phases. See Figure 2 for an example of the specific contingencies connected with responding to S^+^, S^−^ and ambiguous cues.

**Figure 2:**
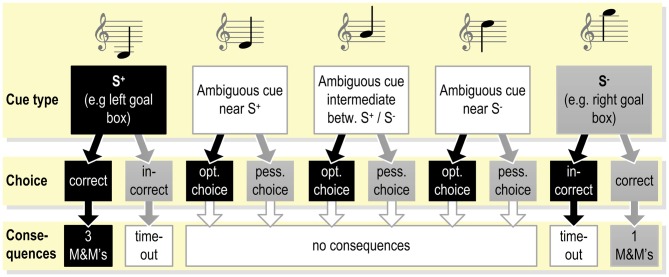
**Example of the exact contingencies connected with responding to the different cues presented during the testing phase (see Figure 1B) in a judgment bias tasks (JBT; programmed consequences of choices as used in Murphy et al., 2013).** Under these or similar testing conditions, the animal already has been trained to respond correctly to the S^+^ and S^−^. As the response to the ambiguous cues does not yield reward, the animal may learn that these cues represent a new class of stimuli.

To successfully perform in a JBT, an animal has to first learn to discriminate between a stimulus (or set of stimuli) that predicts a positive consequence (S^+^) and a stimulus (or set of stimuli) that predicts a negative consequence (S^−^; see Figure 1). Once the animal has mastered this discrimination, at least one ambiguous stimulus is introduced that lies somewhere between the original stimuli, i.e., judgment bias is tested in situations where animals make decisions under ambiguity (Mendl et al., 2011; for an example, see Figure 2).

Two classes of JBTs can be distinguished: Go/Go and Go/No-go tasks. Go/No-go requires suppression of response at S^−^, whereas in Go/Go tasks the animal responds to both types of stimuli with an active response (Murphy et al., 2013).

In both Go/Go and Go/No-go tasks, the animal learns to discriminate between:

1. A *favorable reward* (large reward, immediate reward) and a *less favorable reward* (small reward, delayed reward), or
2. A *positively valenced outcome* (e.g., large food reward) and a *negatively valenced outcome* (e.g., small, less palatable food reward, or food with a bitter taste, no reward). In some cases, the negative outcomes consists of exposure to aversive noise or a frightening stimulus, such as a mild electric foot shock (e.g., Harding et al., 2004), a blower (e.g., Destrez et al., 2013), a dog (e.g., Doyle et al., 2011a), swaying a plastic bag in front of the animal (e.g., Douglas et al., 2012), i.e., consequences on a different modality than the consequences associated with the S^+^.

## Judgment Bias Tasks

Cues used in JBTs are spatial (e.g., Briefer and McElligott, 2013; Destrez et al., 2013; Kis et al., 2015); visual (e.g., Salmeto et al., 2011); auditory (e.g., Murphy et al., 2013), olfactory (e.g., Boleij et al., 2012; see Figure 1), or a combination of different stimulus classes (Douglas et al., 2012). In the latter case it may be difficult to define ambiguous cues and their scaling on the continuum from S^+^ to S^−^. A large variety of testing equipment is used for judgment bias testing such as the home cage (e.g., Boleij et al., 2012), runways (e.g., Salmeto et al., 2011), open fields (e.g., Destrez et al., 2012), or mazes with arms radiating from a start box (e.g., Briefer and McElligott, 2013; see “Supplementary Figure 1”). Owing to the large range of animal species that has been tested in JBTs, species-specific modifications are necessary, concerning the size and layout of the testing arena (if any; e.g., dogs have been tested in their owner’s home: Karagiannis et al., 2015), the stimuli (cues) used; the type of response required (Go/Go, Go/No-go); the type of experimental manipulation used to affect emotion, and the type of consequences as result of the response to a cue (Figure 2; for an example see also Murphy et al., 2013).

## Peak Shift

When considering the use and results of JBTs, it should also be taken into account that basic psychological mechanisms such as generalization gradients and peak shift may play a role in responses to ambiguous stimuli in judgment bias paradigms. Generally, judgment bias paradigms start with the acquisition of a simple discrimination task, in which one stimulus provides a desired outcome (S^+^) and another stimulus provides an undesired outcome (S^−^). Thus, in Go/No-go tasks, responding (in whatever form the task requires) to the S^+^ increases, while responding to the S^−^ decreases. This does not apply to Go/Go tasks, where maintenance of active responding to both the S^+^ and the S^−^ is required.

**Figure 3:**
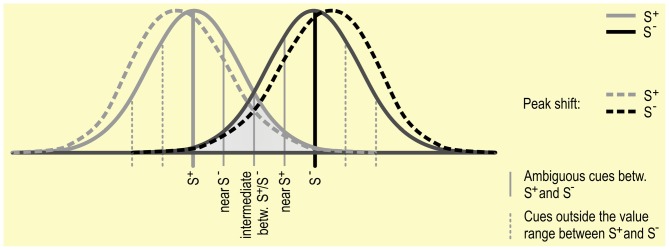
**Discrimination between S^+^ and S^−^: Gradients around S^+^ and S^−^ are depicted as Gaussian distributions.** This distribution reflects the phenomenon of “generalization”, in which stimuli that are more similar to an S are more likely to elicit a similar response. Generalization gradients, and specifically interactions between the gradients around the S^+^ and S^−^, may play a role in responding in JBTs, independent of the judgment bias of the subject. Peak shift, in which the peak of a generalization gradient surrounding an S^+^ shifts away from the S^−^ (see dotted lines), may also influence responding to ambiguous stimuli.

It has been shown in a number of species, including humans, that when animals are trained using one S^+^ and then tested using stimuli similar to but not exactly the same as the S^+^, responses will be highest to the stimuli nearest to the original S^+^. This is called a generalization gradient (see Figure 3; Cheng et al., 1997). The response rate to intermediate stimuli found between an S^+^ and S^−^ is thought to be predicted by the interaction between the two generalization gradients (Hanson, 1957; Kalish and Guttman, 1957, 1959).

The distribution of responses in a generalization gradient around the S^+^ are usually symmetrical if only one stimulus is used. However, if a second stimulus is used (as in most JBTs), the peak of responses to the S^+^ may shift to a cue further from the S^−^ (Ghirlanda and Enquist, 2003), a process termed peak shift. This particularly occurs if the S^+^ and S^−^ are relatively similar to start with. A complicating factor for Go/No-go tasks is that it is difficult to assess whether there is a shift in the generalization gradient surrounding the S^−^, as there is generally a low response rate or no response at all to the S^−^, which predicts an undesired outcome (such as no reward). Results from studies specifically analyzing the responses to the S^−^ seem to indicate that there is also a peak-shift in S^−^ responses (Hanson, 1959), though it is not clear whether this is to the same degree as the influence on S^+^ responding. If peak shift differentially affects generalization gradients surrounding the S^+^ and S^−^, then responding to ambiguous stimuli surrounding the S^+^ and S^−^ may also be differently affected.

For a better understanding of the processes underlying judgment bias, it may be necessary to address the generalization gradients around the discriminative stimuli used. This may include presenting ambiguous stimuli that are outside the values range between S^+^ and S^−^ (see Figure 3) to determine the role that peak shift may play in responding in JBTs (Ghirlanda and Enquist, 2003, p. 20).

## State Vs. Trait

**Figure 4:**
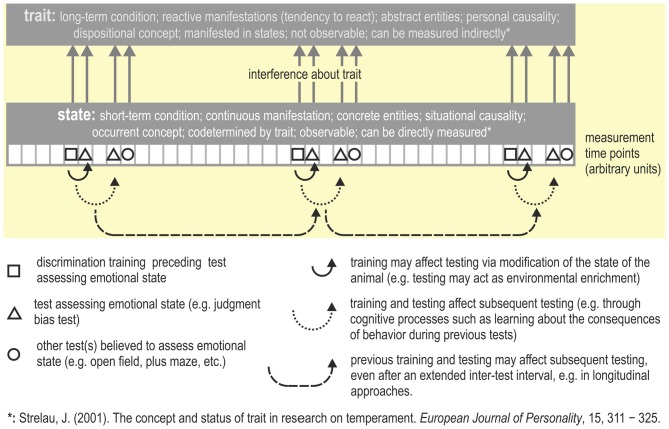
**Measuring trait vs. state.** Repeatedly testing emotional state (e.g., across the lifespan of an animal) may yield information about its emotional trait, i.e., the behavior indicative for a particular trait needs to be repeatable (Carter et al., 2013). In this hypothetical example, the JBT and some other tasks that are believed to assess emotional state are applied multiple times (for an example, see Bethell and Koyama, 2015). Possible consequences of repeated testing are summarized. The description of the concepts trait and state are from Strelau (2001 p. 317, Table 2).

Faustino et al. (2015) suggest that judgment bias may reflect either a state or a trait. However, JBTs have commonly been used to measure the affective *state* of an animal. Modulation of judgment bias through situational or contextual factors which can be observed as within-individual variability (e.g., by providing an enriched living environment, stress, or mood-enhancing drugs) is characteristic of a state. Emotional *trait* can be considered as a constant that is a permanent feature of the individual (Ramos and Mormède, 1998) i.e., may be the expression of a specific phenotype of an individual (Faustino et al., 2015). Similarly, Strelau (2001, p. 311) defined trait as a relatively stable and individual-specific generalized tendency to behave or react in a certain way expressed in a variety of situations (see also Figure 4). In order to assess a trait (which is stable over time), the test(s) used must yield highly replicable results (Carter et al., 2013). A trait thus is considered a permanent characteristic, whereas a state is considered as a transient condition that is only observable at particular moments (see also Fridhandler, 1986; Koski, 2011; Carter et al., 2013).

Anxiety, for example, can be seen as a trait, or a state. Trait anxiety is defined as the intrinsic basal anxiety characteristic of an individual, which does not vary from moment to moment, while state anxiety is defined as the anxiety that an individual experiences at a particular moment in time (Lister, 1990). Trait anxiety is determined by genetic factors, environmental influences and gene by environment interactions. Theoretically animals that have a higher trait anxiety respond to dangerous situations more frequently and with a greater intensity than individuals lower in trait anxiety (Spielberger et al., 1984). More anxious individuals thus have a higher level of trait anxiety and in threatening situations probably also higher levels of state anxiety. The difficulty with state anxiety is that the level of anxiety that is measured depends on both the situation and the level of trait anxiety of the individual. The most reliable measure of the anxiety characteristic of an animal or human thus would be a measure of trait anxiety, and not of state anxiety. In animals it remains to be investigated how to make a distinction between state and trait anxiety. Human studies revealed that judgment bias is influenced by trait anxiety (as measured by questionnaires; see for example Eysenck et al., 1991; Mathews and MacLeod, 1994) as well as state anxiety (Mathews and MacLeod, 1994; Anderson et al., 2012). However, traits are not static; they can change gradually over time under the influence of environmental factors (Strelau, 2001).

For further validation of JBTs, the animal’s behavior during the testing phase should be correlated with behavior in other tasks that are believed to assess emotional states and/or traits. For rodents, these may be tests such as the OF test, the LD test, the elevated PM, the novel object test, and/or the modified hole board, to name a few (e.g., van der Staay et al., 1990; Duncan and Keller, 2011; see Figure 4). In non-rodent species, these tasks may be less adequate and other tasks validated for those particular species must be applied. Some studies have compared JBT performance to another test of emotionality. Judgment bias has been shown to correlate with anxiety in pigs as measured by a novel object test (Carreras et al., 2016). Pessimistic judgment bias was positively correlated with a more fearful response during the novel object test. Rats which laugh when tickled (a confirmed behavioral signal of positive emotional state) have a more positive judgment bias than rats which don’t (Rygula et al., 2012). Destrez et al. (2012) found that lambs treated with an anxiolytic showed a positive judgment bias and were less fearful during isolation and suddenness tests. When studying the responses of laying hens in different tests of emotionality, some correlations were found between measured parameters during a JBT, a novel object test and an anticipation test. However, no clear relationship between the tests was found (Wichman et al., 2012). To test specifically for the effects of emotional traits on judgment bias, examining possible correlations with tasks that measure personality traits is necessary. For example, repeated comparisons of baseline judgment bias of individuals with high vs. low trait anxiety would be valuable. Individual differences in baseline judgment bias have been reported (e.g., Starling et al., 2014). Repeated judgment bias testing has been applied to a small sample of chimpanzees (*n* = 3). Individual differences in judgment bias were found, which remained stable across five test sessions (spanning a time period of 1–2 weeks; Bateson and Nettle, 2015). When a similar study was performed with pigs, no consistent results were found between two test sessions (with a 5 week intermittent period; Carreras et al., 2015). In rats, repeated testing of baseline judgment bias has produced stable results, which correlate with traits such as motivation and sensitivity to stress (Rygula et al., 2013, 2015b).

Though replicability and stability of result is a basic requirement of a trait, this may be difficult to demonstrate empirically. Unfortunately, the order of testing may affect behavior in subsequent tests (e.g., McIlwain et al., 2001; Blokland et al., 2012). This is an observation that may also complicate a correlational approach (e.g., factor analysis) to validating JBTs. To the best of our knowledge, neither the correlations between different tests that are believed to assess emotion with JBTs, nor the effects of repeated (or longitudinal) assessment has yet been studied systematically.

## Cues on One Single Stimulus Dimension Or on Different Stimulus Dimensions?

There are several potential concerns related to the choice of stimulus dimension(s) when preparing a JBT design. There may be variation between animals in their capabilities to differentiate between cues. For example, when using auditory cues, the accuracy of perceiving differences between tones may be different for good and poor listeners in learning the original tone discrimination (see, e.g., Amitay et al., 2005). For olfactory stimuli, it needs to be ensured that mixtures of the S^+^ and S^−^ odors are distinguishable as such, i.e., are not simply regarded as a novel odor, but as intermediates between S^+^ and S^−^ (Dreumont-Boudreau et al., 2006). Differences in ability to discriminate between learned and ambiguous stimuli may similarly affect studies using visual or tactile cues. Additionally, there may be a non-linear relationship between the perception of the originally acquired S^+^/S^−^ and the intermediate stimuli, i.e., due to the sensory capabilities of the species studied, the scaling of cues may not be perceived as intended. For example, what is intended to be an intermediate ambiguous cue may be perceived as having a higher similarity to the S^+^ than to S^−^. The dimension and scaling of the cues used thus may affect performance in JBTs by affecting ambiguity. Therefore, it is important to adjust the dimension and scaling of cues to the species studied (e.g., auditory cues used in several rat studies are adapted to the species’ audiogram (e.g., Enkel et al., 2010; Rygula et al., 2013)).

**Figure 5:**
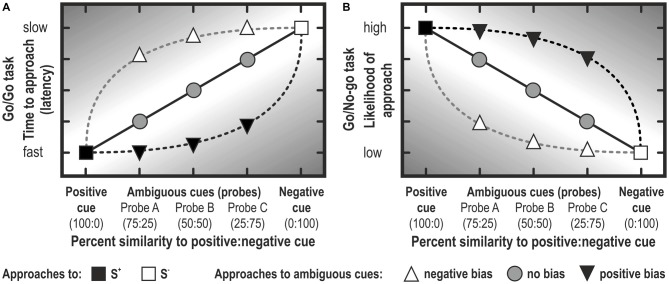
**Judgment bias represented schematically.** Note that the bias is stronger to the extent to which the response lies in the darker area. In Go/Go tasks, the latency to approach a cue is usually analyzed **(A)**, whereas the likelihood (or the proportion of animals in a treatment group) is usually analyzed in Go/No-go tasks **(B)**. Note that, depending on the criterion of defining a no-go response, graphs **(A)** and **(B)** may look quite different (i.e., they are not merely mirrored along the vertical axis; see, for example, Douglas et al., 2012; Gordon and Rogers, 2015). Note that rather often, experiments conceived as Go/No-go tasks report latencies to approach, because animals didn’t learn to suppress responding in the no-go trials (e.g., Bateson et al., 2015).

A number of studies used cues from different dimensions. Although such a methodology might make discrimination between cues easier, they limit the interpretation of JBT results (Nogueira et al., 2015). Graded stimuli on a unidimensional scale allow for the prediction of response patterns (see Figure 5). When different dimensions are used for S^+^ and S^−^, ambiguous cues can no longer be considered as intermediate. For example, Salmeto et al. (2011) used a series of chicken to owl morphs, where the S^+^ was the mirror image of the tested chicken, whereas the S^−^ and the different morphs were silhouettes, printed on cards (the mirror image added an extra dimension to the S^+^, namely movement). When comparing responses to a previous experiment using a chicken silhouette as S^+^, it became clear that the chicks responded differently to the mirror than to printed stimuli, with decreased latencies to respond to the moving mirror images. When the ambiguous stimuli are unrelated to the trained reference cues, there is a risk of measuring response to novelty instead of ambiguity. For example, using wild peccary as subjects, Nogueira et al. (2015) used categorically different auditory stimuli (whistle and horn as S^+^ and S^−^, bell as ambiguous). Ambiguity is characterized by the possibility to interpret a situation or stimulus in two (or more) distinct ways, i.e., in the case of JBTs ambiguous cues can be interpreted as predicting a similar outcome to either S^+^ or S^−^. In order to obtain results which are interpretable as responses to ambiguity, it is suggested to only use cues on a single dimension.

## Loss of Ambiguity within a Small Number of Testing Trials

It is common practice in JBTs to leave test trials (i.e., presentations of ambiguous stimuli) unrewarded (see Figure 2). Such a lack of reward will stand out after extensive training where rewards were always present. This will facilitate learning about unrewarded ambiguous trials (Jamieson et al., 2012). As a result, repeated testing in JBTs could lead to a loss of ambiguity, as the animals will learn to associate the ambiguous stimuli with a specific outcome. This could influence the animals’ subsequent choices during test trials and thereby lead to false conclusions of measured judgment bias (Doyle et al., 2010b). Such possible confounding effects of unrewarded testing trials have been recognized in numerous studies. Brilot et al. (2010) found that their study subjects (starlings) increased the response latencies as testing progressed, while failing to detect a cognitive bias. They concluded that their birds quickly learned that the ambiguous trials were never rewarded and therefore became slower to respond to ambiguous cues. Multiple other studies report a loss of ambiguity as a possible cause for increased response latencies of their study subjects (Doyle et al., 2011b; Sanger et al., 2011; Starling, 2012; Destrez et al., 2014; Starling et al., 2014; Verbeek et al., 2014; Karagiannis et al., 2015). In addition to an increased response latency, Murphy et al. (2013) found that their pigs also decrease the number of optimistic responses with repeated testing. Doyle et al. (2010a) suggested that a loss of ambiguity could even explain why stressed sheep responded more pessimistically than their non-stressed controls.

As mild stress may enhance learning (Mendl et al., 2009), the stressed animals could simply have learned about the lack of rewards during ambiguous trials faster than the control group. Similar conclusions have been drawn in other studies (Destrez et al., 2012, 2013; Scollo et al., 2014). A study dedicated solely to the effect of repeated testing in absence of any experimental manipulations or changes in environment found that sheep develop a reluctance to respond during ambiguous test trials (Doyle et al., 2010b). As there was no explanation for this change in behavior related to a change in their emotional state, an increase in pessimism seems unlikely. Rather, this study supports the notion that animals may learn about the outcome of ambiguous trials with repeated testing and change their responses accordingly.

Several possible solutions to the problem of loss of ambiguity have been suggested. Use of a secondary reinforcer during training and testing was successfully applied in a study by Keen et al. (2014). In addition to a high and low food reward, their bears were also reinforced with a clicker to maintain responsiveness. During ambiguous testing trials, no food rewards were given, but reinforcement with the clicker continued. A secondary reinforcing audio cue has also been used in a study with Rhesus macaques (Bethell et al., 2012). Another measure to reduce learning about the outcome of test trials is a partial reinforcement ratio schedule for training and control trials. For example, Neave et al. (2013) used partial reinforcement of positive trials during training. Although the punishment rate for negative trials remained 100%, they reduced the reward rate for positive trials to 50%. Using this training procedure, their calves learned to have lower expectations of reward during ambiguous trials. Partial reinforcement of training and control trials has also been successfully applied in various other studies (Bateson and Matheson, 2007; Matheson et al., 2008; Bethell et al., 2012; Richter et al., 2012; Neave et al., 2013; Daros et al., 2014; Bateson et al., 2015; Bethell and Koyama, 2015).

The number of learning opportunities about the outcomes of trials during judgment bias testing can also be reduced by minimizing the number of ambiguous test trials. In a study by Vögeli et al. (2014), sheep were subjected to three test sessions of five trials, with each session containing only one single ambiguous trial. No reduction in visits to unrewarded ambiguous probes was reported. Similarly, studies by Rygula et al. (2013, 2015b) report stable judgment bias for their rats by using a relatively small number of ambiguous trials in comparison to positive/negative trials. Although these studies support the notion that limiting the exposure to unrewarded ambiguous probes can prevent loss of ambiguity, this measure would also reduce the number of trials that can be used to estimate effects of experimental manipulations on judgment bias. A reduced number of ambiguous trials may make the JBT results prone to chance findings. Using a between subjects design would at least minimize the number of exposures to ambiguous stimuli per animal (Brilot et al., 2010).

A final suggestion has been to reward ambiguous trials (Murphy, 2015, pp. 185–187; see also Carreras et al., 2015). This was shown to lead to a maintenance of optimistic choosing throughout test sessions, whereas unrewarded test trials lead to a decrease in optimistic choice. However, such a design may still lead to associative learning concerning the outcomes of ambiguous trials, rendering them no longer ambiguous.

Surprisingly, Bateson and Nettle (2015) used no specific measures to avoid loss of ambiguity, yet found no effects of repeated testing in three chimpanzees with respect to the latencies to react to intermediate stimuli. Consequently, they conclude that their JBT is suited for longitudinal assessment of welfare in this species. The authors ascribe their apparent maintenance of ambiguity to the difficulty of their discrimination task (paper cones of 20% vs. 60% gray, with intermediate shades as ambiguous stimuli). However, their very small sample size (*n* = 3) increases the chance of false positive findings (Tversky and Kahneman, 1971). Therefore, repeated testing with a larger sample size would be beneficial to further support their method of preventing loss of ambiguity.

Loss of ambiguity can become a considerable limitation to JBTs, as it renders them ineffective for the detection of changes in affective state (Brilot et al., 2010). Rather, it may cause animals to base their performance on associative learning as testing progresses. This could lead to incorrect conclusions about the effects of experimental manipulations on an animal’s affective state. Therefore, it is recommended to implement precautions against loss of ambiguity, such as the use of a partial reinforcement schedule during training and testing (e.g., Bateson et al., 2015). The specific design of a study will determine which precautions are the most suitable. Also, it is important to exclude loss of ambiguity as a possible explanation for results. This can be done by testing for changes in response to ambiguous stimuli in the absence of experimental manipulation (Neave et al., 2013; Daros et al., 2014).

## Go/No-Go Vs. Go/Go Tasks (Active Choice Tasks)

In an article by Baciadonna and McElligott (2015), of the judgment bias publications reviewed, approximately 70% (22 of the 32 publications) were designed as a Go/No-go task. Of the studies summarized in the Table 1 of “Supplementary Material”, approximately 50% was of the Go/No-go type. Matheson et al. (2008) developed an active choice task for starlings in which the subject must respond to the S^+^ and S^−^ with the same operant behavior (e.g., pecking a key (S^+^) associated with immediate reward, or a key (S^−^) associated with delayed reward). Other variants have since been developed such as an active choice task for pigs, in which responding in the goal box associated with the S^+^ yields a large reward, and responding to the goal box associated with the S^−^, yields a small reward (Murphy et al., 2013).

Theoretically, the main difference between Go/Go tasks and Go/No-go tasks is that in Go/Go or active choice tasks the animal is required to make an active response to both the S^+^ and S^−^, whereas in Go/No-go tasks, the animal is required to suppress a response to the S^−^. In Go/No-go tasks, a cut off criterion is defined to distinguish between the two response classes. Usually, trials in which an animal did not respond within a pre-determined cut-off time are scored as No-go (see Figure 5). Alternatively, the median response time—an approach that determines the cut-off within a preset period empirically—serves to distinguish between Go (latency to respond below median latency) and No-go (latency to respond above median) responses (e.g., Wichman et al., 2012). In both instances, the proportion of animals responding in one of the two classes (Go, No-go) is analyzed. The selected cut-off time may determine the discriminating ability of the test.

The response suppression required for Go/No-go tasks may influence JBT results, as behavioral inhibition is thought to be influenced by emotion (Cyders and Smith, 2007). Moreover, in Go/No-go tasks, No-go responses could be considered an omission to react, rather than a pessimistic response (Guldimann et al., 2015). Active choice tasks circumvent this possible confounding factor of motivation by requiring active responses for both optimistic and pessimistic choices (Hales et al., 2014). As active choice tasks do not require behavioral inhibition and allow for omissions to be measured separately from optimistic/pessimistic responses, they may be more suited for measuring judgment bias than Go/No-go tasks (Murphy et al., 2013). It should be noted that, in practice, a Go/No-go and a Go/Go task are not necessarily mutually exclusive (see Figure 5). If the trial doesn’t stop when reaching the criterion of No-go, but lasts e.g., for two times the criterion duration, then the data can also be analyzed as reflecting active choice responses (see Douglas et al., 2012; Carreras et al., 2015; Gordon and Rogers, 2015).

Many different criteria have been used for mastering the basic discrimination task preceding judgment bias testing. In both Go/Go and Go/No-go tasks similar criteria are used, usually based on accuracy (60 to 90% correct responding to S+ and S−, Anderson et al., 2013; Keen et al., 2014; Rygula et al., 2015a), latency (shorter latencies to respond to S^+^ than to S^−^, Briefer Freymond et al., 2014; Kis et al., 2015), or running speed (faster to S^+^ than S^−^, Karagiannis et al., 2015). Specific to No-go trials, a predetermined number of no-approaches of S^−^ (Sanger et al., 2011) has been used for determining when animals had learned the basic discrimination. The learning criterion must be reached over a predetermined number of training days, trials, or trials within a number of days. All animals that did not reach the criterion within this maximum are excluded from testing with ambiguous cues (e.g., Müller et al., 2012). Sometimes additional criteria, such as that the animal makes no omissions in a fixed number of trials (Anderson et al., 2013), are used.

In some studies, differences between the response to the S^+^ and S^−^, confirmed statistically by Wilcoxon test (e.g., Kis et al., 2015) or Mann-Whitney U-test (e.g., Starling, 2012) were used as criterion. It has not yet been investigated how the learning criterion, i.e., the level of mastering the original S^+^/S^−^ discrimination, affects the sensitivity of subsequent testing with ambiguous cues. It is conceivable that a weak criterion decreases the likelihood to detect a judgment bias.

## Training and Testing in Isolation Vs. Testing in the Social Group

Though not unique for JBTs, testing social animals individually, without direct contact to its group, may increase the stress level and/or decrease the willingness of an animal to learn the task and/or perform the required responses. Extensive habituation and pre-training may be necessary before judgment bias can be individually assessed (see Krasheninnikova and Schneider, 2014). For example, pigs need extensive habituation before they can be trained and tested individually in JBTs (Murphy et al., 2013, 2015).

Training and testing in a group setting is another solution to problems associated with individual testing of social animals. However, group testing is likely also accompanied by methodological issues. To date, only one study has used group training for a JBT. Training white-lipped peccary in isolation was unsuccessful in a study by Nogueira et al. (2015), necessitating training within a group setting. During discrimination training, the animals responded to the S^+^ (approached a food bowl containing rewards) as a group. A similar method was used to acquire correct No-Go responses to the S^−^. The authors mention that extra food rewards were provided when higher-ranking individuals were monopolizing the food bowl. This implies that higher-ranking individuals received more rewards for correct responses than lower-ranking animals, possibly influencing results of discrimination training. Also, differential expectations of reward could have been established, with higher-ranking individuals experiencing a bigger contrast between rewarded and non-rewarded trials. Only animals which reached a criterion level of performance were used for individual judgment bias testing. Another potential limitation of training in a group setting is the difficulty of determining individual performance. Did all animals truly acquire the discrimination between S^+^ and S^−^, or were some individuals simply copying the responses of their group members? This can only be established by acquiring individual results (e.g., by individual training or by evaluating responses to reference tones during individual testing). No systematic comparisons of JBT training and testing in isolation and in social groups have been reported so far. Due to the many potential limitations of training and testing in a group setting, it does not seem likely to be advantageous over individual habituation of social animals.

The effects of (short-term) isolation of social animals, applied as an experimental manipulation of the emotional state preceding judgment bias testing, have been explored in several species. Social isolation affected judgment bias in chicks, with duration of the isolation period having specific effects on JBT performance. A pre-testing isolation period of 5 min induced increased pessimistic responses, while an isolation period of 60 min also decreased optimistic responding (Salmeto et al., 2011; Hymel and Sufka, 2012). For pigs and laying hens, no effect of short-term social isolation on JBT performance was found (Düpjan et al., 2013; Murphy et al., 2013; Hernandez et al., 2015). Only the study by Murphy et al. (2013) mentions habituation of the animals prior to training and testing, possibly explaining why no effect of social isolation was found. When male rats are moved from group housing to individual cages, their rate of optimistic responding decreased (no effect was found for female rats). However, as enrichment and available shelter were also removed when moving the rats, these could have been confounding factors in this study (Barker et al., 2016). Together, these studies suggest that habituation of social animals to the training and testing conditions is sufficient to avoid a confounding influence of stress during testing.

## Excluding Animals that Did Not Pass the Training Preceding Judgment Bias Testing

Many judgment bias studies report the exclusion of animals that failed to reach a required criterion during training (e.g., Starling, 2012; Starling et al., 2014; Verbeek et al., 2014; Bethell and Koyama, 2015; Hernandez et al., 2015). For example, Wichman et al. (2012) reported 10 out of 38 chickens were unable to acquire the discrimination between rewarded and unrewarded trials, in spite of a long training period. These animals could therefore not be subjected to judgment bias testing. Similarly, in group of 18 white-lipped peccary, four adult individuals did not learn the basic discrimination in a Go/No-go auditory discrimination task, and were consequently not tested in the subsequent JBT (Nogueira et al., 2015). In a study by Brajon et al. (2015), only 59% of their 54 pigs completed the training preceding judgment bias testing. Consequently, all results and conclusions from JBTs are based on the study subjects that were capable of learning the discrimination task. If not all animals are able to reach the preset learning criterion, the samples are biased toward “learners”. The larger the proportion of excluded “non-learners” is, the more biased a study is, and consequently, the less the results can be generalized. Development of tests that need less pre-training, e.g., by ensuring the discrimination training is better suited to the natural abilities/behaviors of the studied species, may allow for more animals to participate in subsequent judgment bias testing. Developing discrimination tasks which the studied animals are able to master fairly easily may also prevent selective loss of animals in experimentally manipulated groups. For example, possible effects of stress on learning could lead to animals undergoing a particular treatment (e.g., induced anxiety) being more likely to fail to pass the training phase (Mendl et al., 2009; Conrad, 2010). Increasing the difficulty of discrimination training may increase the likelihood of a larger proportion of non-learners in a specific treatment group.

## Usability for Assessing Animal Welfare

In animal welfare research, JBTs have been applied to a wide range of species that are commonly kept in captivity for a variety of reasons (e.g., production animals such as pigs, Brajon et al., 2015; laboratory animals such as rats, Burman et al., 2008; zoo animals such as Grizzly bears, Keen et al., 2014; companion animals such as dogs, Titulaer et al., 2013). Most of these judgment bias studies have been aimed at investigating the effects of common conditions inherent to life in captivity. For example, the effects of providing environmental enrichment have been studied extensively (e.g., Douglas et al., 2012; Bethell and Koyama, 2015). Also, the effects of common handling procedures have been frequently tested using JBTs, such as dehorning procedures in cattle (e.g., Neave et al., 2013).

According to Bateson and Matheson, (2007 p. 36), “to be practically useful as a measure of how animals feel, cognitive bias needs to be easy to measure in applied settings”. However, many studies needed extensive training on the basic discrimination task before judgment bias could be assessed, decreasing the practical applicability of JBTs as a form of welfare assessment. Additionally, an extensive training period could mask potential detrimental effects of experimental manipulation and is considered one of the most confounding factors in judgment bias test paradigms (Novak et al., 2015). Acting as cognitive enrichment, training could improve the welfare/affective state of the study subjects (Carlstead and Shepherdson, 2000; Puppe et al., 2007; Pomerantz and Terkel, 2009; Zebunke et al., 2011; Guldimann et al., 2015). In spite of negative affect manipulations, this could lead to optimistic responses from subjects (Düpjan et al., 2013). In line with this expectation, in preparation of judgment bias testing, Svendsen (2012) trained farmed mink categorized as fearful or as explorative, to induce a positive affective state. Whereas the fearful mink behaved more explorative at the end of training, an opposite effect of training was found in the mink categorized as explorative: these animals were rated as less positive post-training, possibly due to frustration about the absence of expected rewards during later training sessions. Svendsen et al. (2012, p. 366) cautions that “(…) studies that involve induced affective states and a lot of training of the animals to assess their welfare, such as the cognitive bias method, need to be interpreted carefully as the handling and training has a different effect on animals in different affective states”. Consequently, future research should focus on the question of whether training for a JBT itself modulates the animal’s emotional state.

The sensitivity of judgment bias to detect effects of experimental manipulations on emotions has not yet unequivocally been established. For example, in a study by Keen et al. (2014) the JBT was unable to detect differential effects of environmental enrichment methods in bears. Although behavioral observations showed that the different types of enrichment were valued differently by the bears (some items were interacted with more than others), this did not result in differences in measured judgment bias. It is possible that providing enrichment did not produce a measurable increase in judgment bias because the JBT was not sensitive enough to detect this change in affective state. However, lack of effect to be detected is another possibility, as the bears were already housed in enriched environments. The addition of an extra enrichment item may not have produced a measurable improvement in affective state to begin with. Similarly, another judgment bias study did not discriminate between the welfare of short and long term kenneled dogs (Titulaer et al., 2013). These similar results between animals which are assumed to be experiencing different levels of welfare, could have been considered a result of the lack of sensitivity of JBTs. However, additional measures of welfare (such as behavioral observations and stress hormone levels) did not differ between the groups either. This study shows the importance of validating judgment bias results by comparing them with other measures of welfare.

Reviewing JBTs as tools to assess welfare in farm animals, Baciadonna and McElligott (2015) conclude that these tasks are sensitive to manipulations that induce negative emotions, whereas experimental evidence for sensitivity to manipulations that induce positive emotions is yet weak. This lack of evidence for sensitivity to positive judgment bias could be due to a lack of scientific attention. The majority of judgment bias studies measure the effects of manipulations which are expected to produce a negative affective state. Studies investigating optimistic judgment bias are much less common. For example, Rygula et al. (2012) have shown that laughing rats (displaying a clear behavioral indication of positive affective state) have a more positive judgment bias than rats which don’t laugh when tickled. As improvement of animal welfare relies on both the reduction of negative emotions and the promotion of positive emotions (Boissy et al., 2007), studies aimed at the sensitivity of JBTs to positive emotions are important.

## Usability for Biomedical Research

JBTs have been used as tools for affective state assessment in biomedical research. The majority of these studies have used rodents as their subjects (e.g., mice, Boleij et al., 2012; rats, Kregiel et al., 2016), reflecting the common use of rodents as animal models in biomedical research. In most of the biomedical studies, experimental manipulations were performed prior to testing (Scenario 2 in Figure 1). In such experiments, all study subjects experience similar conditions during training. This is in contrast to many welfare studies, which alter housing conditions, etc., prior to training. Studies which have different conditions for experimental groups prior to or during training, apply experimental manipulations that could affect both the discrimination training preceding judgment bias testing and responding in the JBT proper.

The main aim of judgment bias studies in biomedical research has been to investigate effects of experimental manipulations expected to affect mood in animal models of mood disorders such as depression and/or anxiety. Most of these studies have tested the effects of anxiolytics and/or anti-depressants on judgment bias performance (e.g., Doyle et al., 2011a; Destrez et al., 2012; Hymel and Sufka, 2012; Anderson et al., 2013; Rygula et al., 2014a,b, 2015c).

It is important that results of JBTs are generalizable to other species (e.g., results should simulate the clinical condition of depression/anxiety in humans and inform about the effects of therapeutics believed to modulate these clinical conditions; van der Staay, 2006). If this is not the case, the translational value of judgment bias measurements in non-human animals may be limited. JBTs appear to be a useful tool for studying animal models of depression and anxiety. Multiple studies have found responses comparable to those found in human studies of judgment bias (Enkel et al., 2010; Salmeto et al., 2011; Hymel and Sufka, 2012; Richter et al., 2012; Papciak et al., 2013; Kloke et al., 2014; Rygula et al., 2014a, 2015a; Kregiel et al., 2016).

JBTs appear to have particular potential to differentiate between anxiety disorders and depression. Although both mood disorders result in a negative affective state, they produce different response profiles in the JBT. In humans, depression is expressed by a decrease in optimistic responses combined with an increase in pessimistic responses. Individuals suffering from anxiety only display increased pessimistic responding (MacLeod and Byrne, 1996; Miranda and Mennin, 2007). These findings have been replicated in judgment bias studies using a chick model of anxiety and depression (Salmeto et al., 2011; Hymel and Sufka, 2012). Chicks in an anxiety-like state displayed more pessimistic behaviors in response to ambiguous aversive cues (i.e., ambiguous cue near S^−^) and to intermediate ambiguous cues. Chicks in a depression-like state behaved similarly, but in addition displayed less optimistic behaviors in response to ambiguous cues near the S^+^. These results highlight the importance of using a spectrum of ambiguous stimuli, ranging from near-negative to near-positive. Different same-valence affective states (such as depression and anxiety) may produce different responses to these different forms of ambiguous cues (Kloke et al., 2014).

The translational value of biomedical judgment bias studies is of particular importance, as results are used for comparison with humans and/or other model species. Therefore, differences between species in baseline responding during a JBT require attention (e.g., is a pessimistic response caused by induction of a negative affective state or by a trait of the studied species, see “State vs. Trait” Section). Several studies have reported a baseline judgment bias of their study subjects. Using test designs with reward and punishment, both rats and BALB/c mice displayed a baseline negative judgment bias. They showed punishment avoidance during ambiguous cue presentation (Boleij et al., 2012; Anderson et al., 2013; Rygula et al., 2015c). These findings could have been a direct result of the test design and comparisons to studies using a discrimination task based on favorable/less favorable reward would be valuable. One study found a baseline positive judgment bias in rats and ascribed this optimism to the favorable testing conditions, i.e., the possibility of food reward and exploration of novel environment, further indicating effects of test design on baseline judgment bias performance (McGuire et al., 2015). Rygula et al. (2014b) reported a difference in baseline judgment bias between groups of rats used for separate experiments, citing this as a possible reason for differences in results found after experimental manipulation. This finding implies that even within-species/strain differences in baseline judgment bias are a possibility that needs to be taken into account. In support of this argument, several studies mention individual variation in judgment bias as a possible influence on their results (Verbeek et al., 2014; Kis et al., 2015).

Biomedical studies have commonly used JBTs to assess effects of drug treatments. When tested drugs have side effects, this could influence behavior in JBTs. One common pharmacological side effect is a reduction or increase of appetite. Most JBTs use food as a reward for correct responses to S^+^, with numerous studies also using (less palatable) food as punishment predicted by S^−^. When treated study subjects experience a decrease in appetite, their performance of optimistic behaviors (i.e., collecting a food reward) may be reduced independent of their affective state. Two studies have mentioned a decrease in appetite as a possible side effect of drug treatments and both used food reinforcers as part of their experimental design (Anderson et al., 2013; Rygula et al., 2014b). An increase in appetite after treatment with the anxiolytic diazepam was discussed as a possible explanation for the observed negative judgment bias (pessimistic responses required the intake of food items with low palatability, see Boleij, 2013). Observing animals’ responses to S^+^ and S^−^ could provide an indication of appetite-related side effects affecting treated subjects. For instance, when responding during food-rewarded positive trials remains high, it is unlikely that a change in appetite is responsible for a change in responding during ambiguous trials. Using an alternative to food reinforcers will rule out treatment-induced differences in food motivation altogether (e.g., Kloke et al., 2014). When food reinforcers have been used, the possibility of side effects of treatment on the consummatory behavior of study subjects should be ruled out (Mendl et al., 2009). There are other common side effects of pharmacological manipulation to reckon with that potentially influence behavior in JBTs (e.g., locomotor activity, lethargy). Doyle et al. (2011a), for example, examined possible side effects affecting the results of dose response trials in a JBT by examining the behavior and physiology of their animals in simple tests of food motivation, reactivity and locomotion.

## Conclusions and Future Directions

JBTs may be suited to assess the emotional state of an animal. Provided that judgment bias can be repeatedly tested in the same animal over a longer time period (Q.E.D., see “Discussion” above, “Loss of Ambiguity Within a Small Number of Testing Trials” Section), it may also be suited to assess emotional trait in animals.

Judgment bias can be tested in a very broad range of species, from insects to humans, i.e., may allow comparison between species, and may be suited for translational research. There is a large variation in test equipment and testing procedures between and within species (for a recent review see Bethell, 2015; see also “Supplementary Material, Table 1”). A huge variation in criteria is applied for mastering the basic discrimination task. Also, a large range of computational and statistical methods to analyze the judgment bias data has been used. Recently, Gygax (2014) reviewed these methods and gave recommendations, which already have been critically commented upon (Bateson et al., 2015). This gamut of statistical analyses hampers comparisons within and between studies and species.

JBTs have been used in the field of animal welfare research and in biomedical research. These tasks need to be further developed and adapted to the species of animals used and the research questions to be addressed. In welfare assessment studies, modifications may include the applicability under non-laboratory conditions, testing of social animals in groups and increase of the efficacy to train animals on the basic discrimination task. Owing to the extensive training preceding judgment bias testing, this task appears to be less suited for routine monitoring of animal welfare.

In biomedical research, a lengthy training period, preceding testing with ambiguous stimuli, may be less of a concern, as drug treatments usually start after completion of learning the basic discrimination between S^+^/S^−^. However, the problem that ambiguous stimuli may lose their ambiguity very quickly, enabling collection of data in a few trials only, needs to be solved. Also, we need to assess whether the task is suited for repeated testing in a longitudinal design (see also Figure 4).

Many open questions, addressed in the present article, still need to be answered before JBTs may be considered as a validated, useful tool in the toolbox of researchers interested in measuring animal emotions in both the context of animal welfare studies and biomedical studies.

## Author Contributions

All authors contributed to writing the manuscript. All authors have approved the final version of the manuscript.

## Conflict of Interest Statement

The authors declare that the research was conducted in the absence of any commercial or financial relationships that could be construed as a potential conflict of interest.
