# Supplementary material for: Making Decisions under Ambiguity: Judgment Bias Tasks for Assessing Emotional State in Animals
Source: Front Behav Neurosci. 2016 Jun 9;10:119. doi: 10.3389/fnbeh.2016.00119 (PMC4899464; doi:10.3389/fnbeh.2016.00119)
Supplement: Supplementary file 1 [file Image_1.pdf]

Supplementary Material

Making decisions under ambiguity: judgment bias tests for assessing emotional state in animals

Roelofs, S.*, Boleij, H., Nordquist, R.E. & van der Staay, F.J.

*** Correspondence:** Sanne Roelofs: s.roelofs@uu.nl

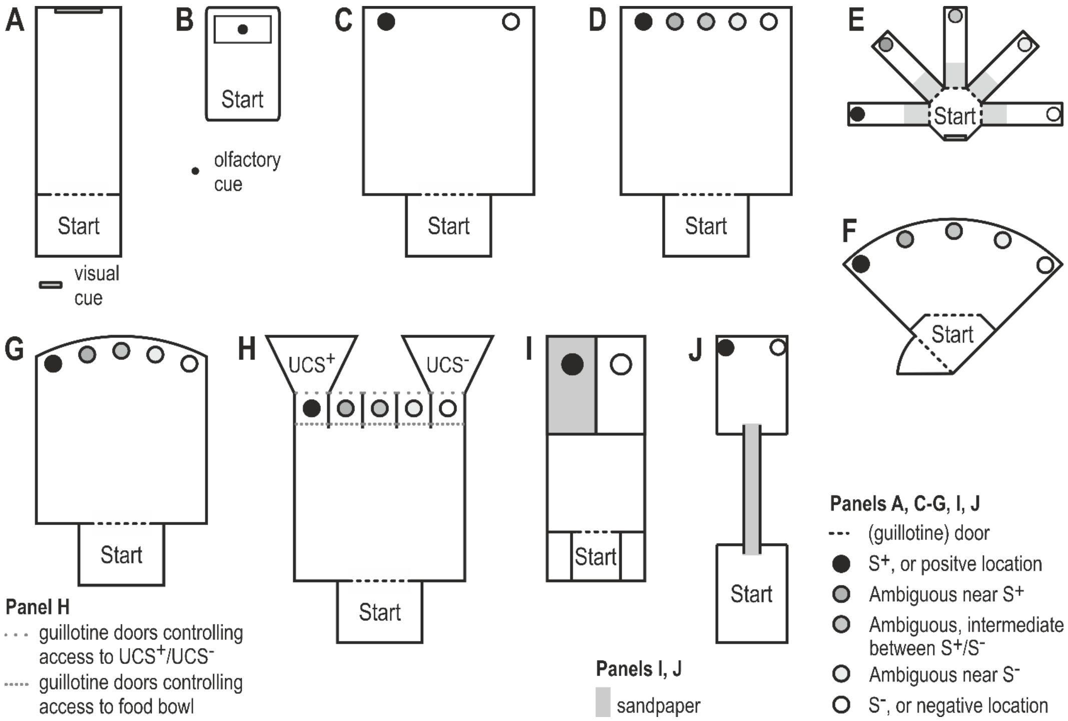

Supplementary Figure 1. Simplified schematic representation of test arenas for assessing judgment bias in animals. (**A**): runway (e.g. Burman et al., 2011; Salmeto et al., 2011); (**B**): rodent home cage (Boleij et al., 2012); (**C**): two choice box (e.g. Murphy et al., 2013); (**D**), (**F**): arenas for presenting cues in spatially distinct places (e.g. Destrez et al., 2012; Doyle et al., 2010; Düpjan et al., 2013), (**E**): maze to present cues in spatially distinct places (e.g. Briefer and McElligott, 2013; Richter et al., 2012); note that in some studies, only three positions are used, namely the S^+ and S^- position, and the position intermediate between S+ and S- (e.g. Carreras et al., 2015); (**G**): same as (**D**), but stimuli are equidistant from start area (Titulaer et al., 2013); (**H**): similar to (**D**), but guillotines control access to the food bowls, and guillotine doors in the rear control access to UCS^+ (e.g. conspecifics) and UCS^- (e.g. a dog) (Verbeek et al., 2014a, 2014b); (**I**): apparatus for testing mice using tactile stimuli (Novak et al., in press); (**J**): apparatus for testing rats using tactile stimuli (Barker et al., in press). Note: not drawn to same scale. Size of the testing equipment depends on size of the species tested. Judgment bias task in arenas as depicted in (**A**), (**B**) and (**C**) normally use non-spatial cues, although the basic discrimination training in arena (**C**) also involves a spatial component. In (**D**) – (**H**), only one location is presented per trial during testing.
