# Supplementary material for: Making Decisions under Ambiguity: Judgment Bias Tasks for Assessing Emotional State in Animals
Source: Front Behav Neurosci. 2016 Jun 9;10:119. doi: 10.3389/fnbeh.2016.00119 (PMC4899464; doi:10.3389/fnbeh.2016.00119)
Supplement: Supplementary file 2 [file Table_1.pdf]

Supplementary material

Making decisions under ambiguity: judgment bias tests for assessing emotional state in animals

Roelofs, S.*, Boleij, H., Nordquist, R.E. & van der Staay, F.J.

* **Correspondence:** Sanne Roelofs: s.roelofs@uu.nl

Table 1. Summary of judgment bias experiments using animals as subjects. Note that, if a study encompassed different behavioral tests, only the judgment bias tasks are included in this table.
Abbreviations:
Sex: f, female; m, male; When (1, 2) refer to when experimental manipulations were performed that are believed to affect emotion (see scenario 1 and scenario 2 in Figure 1); n.a.: not applicable; Test arena: A – H refer to arenas depicted in Supplementary Figure 1; S^-/S^+, conditioned stimuli used: a, acoustic; o, olfactory; s, spatial; t, tactile; v, visual; d, different dimensions; Ambiguous stimuli: a, acoustic, o, olfactory, s, spatial, v, visual, d, different dimensions. In addition, number of different ambiguous stimuli is shown between parentheses; Go/No-go: Go/No-go task; Go/Go: active choice task; Welfare: indicates whether animal welfare was explicitly addressed.

Species (strain/breed)	Sex	Experimental manipulation(s)	When	Test-arena	S^-/S^+	Ambiguous stimuli (nr.)	Training	Testing	Go/ No-go	Go/Go	Welfare	Effect(s) of experimental manipulation(s)	Reference
Rats, Lister-hooded	m	Pharmacology (experiment 1): acute diazepam, reboxetine and fluoxetine, 3 doses per drug	2	[1]	a; tones; S^+/S^- counterbalanced 2 or 8 kHz	a(3); tones: 4,5 and 6 kHz	correct left or right lever press following S^+/S^- results in reward (food pellet)/avoidance of punishment (foot shock). Learning criterion: accuracy>60% and no omissions in 10 consecutive trials for 2 days	Randomized Latin square design, all animals received all treatments on separate days. Exposure 40 reference tones (negative and positive) and 20 mid-point probe tones (4, 5 and 6 kHz)		✓		Reboxetine reduced probability of reward response (negative bias) and increased omissions. Fluoxetine and diazepam had no effects.	(Anderson et al., 2013)

[1] Tone discrimination task in a Skinnerbox

Species (strain/breed)	Sex	Experimental manipulation(s)	When	Test-arena	S^-/S^+	Ambiguous stimuli (nr.)	Training	Testing	Go/ No-go	Go/Go	Welfare	Effect(s) of experimental manipulation(s)	Reference
Rats, Lister-hooded	m	Pharmacology (experiment 2): Two baseline training weeks (after acute drug testing, exp. 1). Then 2 groups: chronic fluoxetine and saline for 3 weeks.	2[2]	[1]	a; tones; S^+/S^- counterbalanced 2 or 8 kHz	a(1); tone of 5 kHz	Re-baselined with training after experiment 1. correct left or right lever press following S^+/S^- results in reward (food pellet)/avoidance of punishment (foot shock). Learning criterion: accuracy>60% and no omissions in 10 consecutive trials for 2 days Then training on Mon, Wed and Thu and exposure to ambiguous tone in trials on Tue and Fri	Testing with mid-point probe on Tue and Fri. 35 reference tones (positive and negative) and 30 mid-point probe tones Testing took place 5 weeks (1 week pre-treatment, during 3 weeks chronic treatment and 1 week post testing)		✓		Fluoxetine treatment increased probability of reward response over time (positive bias) although also the control group shows this effect to a lesser extend	(Anderson et al., 2013)
Common marmosets (*Callithrix jacchus*)	f, m	Rearing conditions; Condition 1: family-reared twins; Condition 2: family-reared animals from triplet litters, where only two animals remained; Condition 3: family-reared triplets that received supplementary food	1	3	v; wooden tubes; S^-: 2 cm high; S^+: 15 cm high, or vice versa; A piece of rusk, hidden under the S^+ tube, served as reward	v(3): 5.5, 8.5, and 11.5 cm high tubes	S^+ and S^- were presented one ate a time. A go response to the S^+ was rewarded, a no-go response to the S- was unrewarded, a go response to the S^- was punished with a 5-second time out; Criterion: 80% correct responses to S^- and S^+ over 3 successive days, with S^+ and S^- presented in random order	Three test sessions with the S^-, S^+, and with the three ambiguous stimuli. Number of no-go responses to the ambiguous stimuli was recorded. Sessions in which the marmoset made < 80% correct responses to S^+ and S^- were omitted.	✓		✓	No effects of rearing condition and gender on acquiring the discrimination task preceding judgment bias testing; no effects of rearing conditions on responding to the ambiguous stimuli. Triplets that had received supplementary food showed less go responses the intermediate ambiguous and the S^+ stimulus than marmosets from the other two conditions.	(Ash and Buchanan-Smith, 2016)
Sprague Dawley rats	f, m	Rats from standard group housing in open top cages were moved to individual metabolic cage housing; controls stayed in their open-top cages	2	J	t; rough (P80) versus smooth sandpaper (P220)	t(1); sandpaper of intermediate grade (P180).	One stimulus, rough sandpaper, was associated with a high-positive reward (chocolate), whilst the other, smooth sandpaper, was associated with a low-positive reward (cereal), or vice versa. One of the two food bowls in the goal box was associated with chocolate, the other with cereal reward. Rats were trained until they had learned the association between sandpaper, food bowl and reward	Rats were moved to the individual metabolic cages or stayed in their open-tp cage, were trained 3 more days, and were then tested for 5 days with intermediate (sandpaper P180), unrewarded trials and with the originally learned associations.		✓	✓	Initiating foraging behavior was longer in trials with the less preferred than with the preferred reward. The number of optimistic decisions over 5 days was larger in males that remained in standard housing than moved to the metabolic cages. This was not observed in females. Male rats moved to metabolic cages had a significantly longer time to initiate foraging for each probe trial than female rats. This difference was not observed between male and female rats that stayed in standard housing	(Barker et al., in press)
European starlings (*Sturnus vulgaris*)	f, m	Housing enrichment	1	A	v; lid of petri dish; S^+/S^-: counterbalanced white or dark grey	v(3); 20, 40 and 60% grey	S^+/S^- is associated with palatable/ unpalatable mealworm. Response: Flip lid of petri dish Learning criterion: significant difference between white and dark lids flipped over 3 consecutive sessions	Test session with 4 reference cues (positive and negative) and 20 probe cues	✓		✓	Starlings that experienced enrichment before standard housing conditions had a shift towards a more negative bias, while judgement biases were not different the other way around	(Bateson and Matheson, 2007)

[2] Multiple testing during chronic treatment

[3] Visual discrimination using different sizes of wooden tubes

Species (strain/breed)	Sex	Experimental manipulation(s)	When	Test-arena	S^-/S^+	Ambiguous stimuli (nr.)	Training	Testing	Go/ No-go	Go/Go	Welfare	Effect(s) of experimental manipulation(s)	Reference
Chimpanzees	f, m	Individual differences between chimpanzees (2 males, 1 female)	1	[4]	v; paper cone; S^+: 20% grey, S^-: 60% grey	v(3); 30, 40 and 50% grey paper cones	Pale grey cones concealed a peanut reward (go-response); dark grey paper cones concealed no reward (no-go); 107-240 acquisition trials.	5 test phases, each preceded by a choice phase. In choice phases, both the S^+ and S^- were presented, with the position randomized (left/right). Progression to test phase if 18 or more choices for POS from a total of 24 trials Test consisted of 3 times 3 ambiguous and 8 S^+ and 8 S^-. Latency to touch the cone was recorded The go response was never rewarded.	✓		✓	Chimpanzees showed differences in speed to touch the cones. In the testing phase, this latency served as covariate. The chimpanzees differed on the latencies to touch ambiguous stimuli; Repeated testing did not affect the response to the ambiguous stimuli. Correlation with rank: highest rank: least pessimistic, lowers rank: most pessimistic.	(Bateson and Nettle, 2015)
Worker honeybees (*Apis melifera carnica*)	f	60 seconds shaking	2	[5]	o; S^+/S^-: 2 different odors counterbalanced 1:9 or 9:1 (1-hexanol and 2-octanone)	o(3); odors in different proportions (3:7, 1:1, 7:3).	2 different odors were conditioned with palatable (sucrose) or unpalatable solution (quinine). One session of 12 trials, pseudorandom presentation.	On the same day as training, unreinforced test trial with 5 odors; the 2 reference odors and 3 ambiguous odors A binary response was measured as outcome variable: whether or not the honeybee extended its proboscis	✓		✓	Agitated honeybees are more likely to classify ambiguous cues with punishment (negative bias), as they extended their proboscis less towards the ambiguous and negative stimulus	(Bateson et al., 2011)
European Starlings (*Sturnus vulgaris*)	f, m	Developmental telomere attrition (possible candidate indicator of somatic state), high vs low competition nests, and number of heavier competitors in the nests	1	A	v; lid of petri dish; S^+/S^-: counterbalanced light (20%) or dark grey (60%)	v(3); lid of petri dish: 30, 40 and 50% grey	The S^+ revealed a palatable mealworm, the S^- revealed an unpalatable mealworm injected with quinine. Criterion: S^+ latency > S^-latency per starling Mann-Whitney U-test). One 16-trials session on a partial reinforcement schedule to slow down extinction during judgment bias testing.	Four daily sessions of 18 trials, with no reinforcement in ambiguous-cue trials, and partial reinforcement in S^+ and S^- trials when the starlings were on average 96 days old	✓		✓	Starling from high competition nests with heavier competitors chose ambiguous stimuli slower. Birds with greater developmental telomere attrition showed chose ambiguous stimuli faster. Authors discuss whether increased reward expectancy reflects a more positive affective state.	(Bateson et al., 2015)
Rhesus macaques (*Macaca mulatta*)	m	Environmental enrichment vs. post- health check	2	[6]	v; line on screen; S^+/S^- counterbalanced short and long line	v(3); lines of intermediate length	S^+ associated with 40% probability of reward (2 food pellets and tone) if screen was touched and S^- with a burst of white noise if touching screen. Training criterion: ≥80% correct responses (>70% correct for both S^+ and S^-)	6 test sessions. Three blocks per test session; 75% correct responses to reference cues means progression to the second block (24 S^+, 24 S^- and 18 ambiguous cues), third block control (10 S^+ and 10 S^-). Latency and frequency of responses recorded.	✓		✓	More reward related responses towards cues closer to the positive cue in enriched animals in comparison with after the health check.	(Bethell et al., 2012)

[4] Visual discrimination using paper cones of different shades of grey

[5] Proboscis extension towards odor

[6] Discrimination task on touchscreen

Species (strain/breed)	Sex	Experimental manipulation(s)	When	Test-arena	S-/S+	Ambiguous stimuli (nr.)	Training	Testing	Go/ No-go	Go/Go	Welfare	Effect(s) of experimental manipula-tion(s)	Reference
Hamster (*Mes-ocricetus Auratus)*	m	Effects of adding or removing enrichment items from an enriched environment. Both orders of environmental condi-tions were presented	2	D	s;<br>S^+/S^- drinker positions (left or right) in a testing arena	s(3); drinker positions in between location S^+ and S^-	The drinker positions were condi-tioned with palatable (sucrose or unpalatable solution (quinine), using a variable reinforcement schedule. Criterion of learning: mean latency to approach S^+ < latency to ap-proach S^-	During stay in environment with added or removed enrichment items, 3 sessions with S+, S- and 3 ambiguous locations. Drinkers were empty.	✓		✓	No effects of the treatments on latency to approach S^+ and S^- were found<br>Adding enrichment induced a positive shift, removal induced a negative shift in proportion of responses towards the near-positive and near-negative cues. No effects were found on responses to the middle probe.<br>No relationship with standard tests of emotionality (open field, light-dark test, approach to novel object) that were performed after judgment bias tests).	(Bethell and Koyama, 2015)
Mice, 129P3/J and BALB/cJ	m	Strain comparison: no manipula-tion (experiment 1)	—	B	o;<br>S^+/S^-: counter-balanced vanilla or apple odor placed in odor cup	o(3): 15/85, 50/50 and 85/15% odor solutions	Odor cup presented with a palatable (positive) or unpalatable (negative, quinine flavored) almond piece. Short training 3 positive and 1 negative trial.	Different groups tested on positive, negative and probe cues; all cues presented with a normal almond piece, approach times measured.	✓			Strain difference in performance of the odors conditioning task. BALB/c mice discriminate between the odors and seem to show intermediate reactions towards the ambiguous cues.	(Boleij et al., 2012)
Mice, BALB/cJ	m	White light vs. dark light testing (experiment 2)	2	B	o;<br>S^+/S^-: counter-balanced vanilla or apple odor placed in odor cup	o(1): 50/50% odour solution	Odor cup presented with a palatable (positive) or unpalatable (negative, quinine flavored) almond piece. Short training 5 positive and 3 negative trials.	Different groups tested on positive, negative and the probe cue. One group tested under a white lamp. All cues presented with a normal almond piece, approach times measured	✓			Latencies to approach the ambiguous cue were comparable to that towards the negative cue, white light caused a general increase on latency times to approach all cues	(Boleij et al., 2012)
Mice, BALB/cJ	m	Diazepam effects (0, 1 and 3 mg/kg)	2	B	o;<br>S^+/S^-: counter-balanced vanilla or apple odor placed in odor cup	o(1): 50/50% odour solution	Odor cup presented with a palatable (positive) or unpalatable (negative, quinine flavored) almond piece. Short training 13 positive and 12 negative trials.	Three groups (0, 1, or 3mg/kg diazepam) tested on positive, negative and the probe cue (2 of each) in one test session.	✓			A subgroup of animals tended to show a negative judgement bias. However, diazepam increased the tendency of mice to eat the bitter tasting almond piece, suggesting that reactions might have been influenced by the effect of diaze-pam on taste and palatability.	(Boleij, 2013)

Species (strain/breed)	Sex	Experimental manipulation(s)	When	Test-arena	S^-/S^+	Ambiguous stimuli (nr.)	Training	Testing	Go/ No-go	Go/Go	Welfare	Effect(s) of experimental manipulation(s)	Reference
Pigs, Yorkshire x Landrace) x Duroc	f, m	Gentle, rough, or minimal handling starting after weaning till the end of cognitive bias testing	1	[7]	a; low and high pitched tones (2093 Hz and 32.7 Hz) predicted a reward (P, S^+) or punishment (N, S^-).	a(3)	Both US were given in a chamber which opened after playing the tone. Responding to S^- yielded different aversive stimuli such as water spray, air puff, falling tennis ball; response to S^+ yielded two pieces of cereals. Training to the criterion of 5 out of 6 trials correct (at presentation of 3 rewarded (positive, approach) and 3 punished (negative trials, avoid); only 32 of the 54 piglets reached criterion	Testing was performed in the presence or absence of a human observer -- the handler in the gentle and rough handling group, an unfamiliar person in the minimal handling group	✓		✓	Piglets in the gentle handling group showed a larger percent approaches at presentation of the ambiguous stimulus intermediate between S^- and S^+; Presence of a human observer reduced the time in contact with the trough, irrespective of the experienced handling (gentle, rough, minimal)., but did not affect judgment bias per se.	(Brajon et al., 2015)
Goats, different breeds	f, m	No manipulation, groups based on history of the goats in the sanctuary (poor welfare or control)	1	E	s; S^+/S^-: Left or right arm in a radial arm maze, counterbalanced	s(3); three arms in between the S^+ and S^-	Left or right arms of radial arm maze, associated with food or no reward. 3 positive and negative trials per day for 3 days.	Two test days, exposure to 2 times the reference cues and one time each ambiguous cue	✓		✓	Sex differences in judgement bias. Females rescued from poor welfare showed more optimistic and less pessimistic responses while there were no differences found between the male groups.	(Briefer and McElligott, 2013)
Horses, three Franches-Montagnes, threeTrotters and six Swiss half-bred	f	Training using positive reinforcement (PR; food reward after correct behavior) or negative reinforcement (NR; cessation of uncomfortable stimulus after correct behavior)	2	D	s; S^+: bucket with food on one side of paddock S^-: empty bucket on opposite side of paddock	s(3): buckets in between S^- and S^+ location	Training (3 days) to associate one location with food reward and other location with no reward. 6 (3 of each) trials/day. Training criterion: significant difference in latency to approach between both.	One session of 7 trials/day, 2 testing days. 2 S^+, 2 S^- and 3 ambiguous trials (1 for each ambiguous location), ambiguous trials in random order with S^+ and S^- interspersed. Ambiguous trials unrewarded.		✓	✓	PR mares went slower to the negative location and adjacent ambiguous location, indicating more pessimistic responding. No differences were found for any of the other locations.	(Briefer Freymond et al., 2014)
European starlings (*Sturnus vulgaris*)	f, m	Cage enrichment and removal of enrichment	2	A	v; lid of petri dish; S^+ dark grey (60%), S^- white (0%)	v(3); intermediate grey (15, 30 and 45%)	If birds made the correct response, it was rewarded with three mealworms (S^+) or one mealworm (S^-). The incorrect response yielded not reward. Learning criterion: significant difference from chance level over three consecutive days (binomial test).	One session of 15 trials per day. 5 times each reference cue reinforced, two times unreinforced and three times ambiguous probe cues		✓	✓	Change in environmental conditions caused no differences in responses towards the ambiguous cues. However, stereotyping starlings showed more pessimistic responses.	(Brilot et al., 2010)
European starlings (*Sturnus vulgaris*)	f, m	Auditory threat/alarm or sparrow hawk calls during testing	2	A	v; aversive stimulus: eyespots, positive stimulus: no eyespots	v(1); ambiguous (partly clouded) eyespots	No training involved, natural aversive cues	Eight sessions per bird with 3 trials, each stimulus used. Exposure 2 times to the different auditory cues while tested	✓		✓	Alarm calls and white noise induced higher freezing and also the eyespots were aversive, since latency times towards the food were increased. No differences towards the ambiguous cues found.	(Brilot et al., 2009)

[7] Box with one food trough, equipped with air spray gun, water spray gun and a release mechanism to drop a tennis ball in the back of the piglet

Species (strain/breed)	Sex	Experimental manipulation(s)	When	Test-arena	S^-/S^+	Ambiguous stimuli (nr.)	Training	Testing	Go/ No-go	Go/Go	Welfare	Effect(s) of experimental manipulation(s)	Reference
Rats, Lister Hooded	m	Cage enrichment (1 week after training)	2	8	t; S^+/S^-: fine or course sandpaper in tunnel	t(1): intermediate grade of sandpaper	Rats move through a tunnel covered with S^+ or S^-. S^+ results in a high value reward (1/2 chocolate drop) if correct choice is made to go to scented cup on left/right. S^- results in a lower value reward (cheerio) if correct choice is made in a scented cup on the right/left. Learning criteria: ¾ trials correct over 5 days	Testing in one week, 5 days of 4 trials. One probe trial: presentation of intermediate sandpaper with no rewards present		✓	✓	Moving to an enriched environment induced a more optimistic choice bias	(Brydges et al., 2011)
Rats, Lister Hooded	f, m	Juvenile stress	1	[8]	t; S^+/S^-: fine or course sandpaper in tunnel	t(1): intermediate grade of sandpaper	Rats move through a tunnel covered with S^+ or S^-. S^+ results in a high value reward (1/2 chocolate drop) if correct choice is made to go to scented cup on left/right. S^- results in a lower value reward (cheerio) if correct choice is made in a scented cup on the right/left. Learning criteria: ¾ trials correct over 5 days	Testing in one week, 5 days of 4 trials. One probe trial: presentation of intermediate sandpaper with no rewards present		✓	✓	Control animals displayed a pessimistic choice bias, while animals that received juvenile stress were more optimistic in their choices	(Brydges et al., 2012)
Rats, Lister Hooded	m	Removal of enrichment in one group during whole experiment, other group remained enriched	1	F	s; S^+/S^- place of goal pot presentation, one side of arena/other side of arena	s(3): three locations in between S^+ and S^-	S^+ goal pot contains 2 food pellets, presentation of S^- is unrewarded, contains 2 inaccessible food pellets. 12 (6 of each) trials/day. Training criterion: significant difference in latency between both.	3 days of testing. Each ambiguous cue (3 in total) presented once each day in between the conditioned cues. 13 trails/day	✓		✓	Unenriched rats approached the probe cue nearest to the negative cue more slowly than enriched rats indicating a negative judgement bias.	(Burman et al., 2008)
Rats, Lister Hooded	m	high (H) or low light (L) training and/or subsequent testing (four conditions: HH, HL, LH, LL)	2	E	s; S^+/S^-: opposite locations in a radial arm maze	s(3): three arms in between S^+ and S^-	Left or right arms of radial arm maze, associated with goal pot with food pellet (S^+) or quinine food pellet (S^-). Learning criterion: significant difference in latency between both.	3 days of testing. Each ambiguous cue (3 in total) presented once each day in between the conditioned cues. 13 trails/day	✓		✓	Only a change in light conditions had effects on latencies in the probe trials. HL rats ran faster in the probe trials than LH rats. LH rats a more negative judgement bias than HL rats.	(Burman et al., 2009)
Dogs, Beagle	f	'Post-consumption' treatment (a rewarding event prior to testing) vs. 'neutral' treatment (no treatment prior to testing)	2	A	v; S^+/S^- dark and light shade of grey	v(3); intermediate. shades of grey	Approach goal box at S^+ (rewarded with food)/ avoid approach. S^- (unrewarded); criterion: approach S^+ faster than S^- for six consecutive trials	Three test days, test sequence of 15 trials with ambiguous stimuli interspersed between S^+/S^- , ambiguous stimuli unrewarded	✓			'Post-consumption' group responded slower to middle ambiguous stimulus	(Burman et al., 2011)

8 Tactile stimuli choice task, tunnel with course or fine sandpaper

Species (strain/breed)	Sex	Experimental manipulation(s)	When	Test-arena	S^-/S^+	Ambiguous stimuli (nr.)	Training	Testing	Go/ No-go	Go/Go	Welfare	Effect(s) of experimental manipulation(s)	Reference
Pigs, Large White Landrace halothane Gene−RYR1−free sows with Pietrain heterozygous boars	m	Testing of cognitive bias twice, with an inter-test interval of 5 weeks; categorization of pigs as having a positive, neutral, or negative cognitive bias, based on first cognitive bias testing	1	D	s; S^- bucket location with inaccessible reward; S^+ bucket location with accessible reward	s(1); bucket intermediate between S^+ and S^-	Approach bucket S^+ (rewarded with chopped apple), avoid approach S^- bucket (inaccessible chopped apple); criterion: approach S^+ faster than S^- for six consecutive trials	After 14 training sessions and 4 reminder sessions, pigs were tested in one cognitive bias session. After a 5-week interval, pigs received 4 training, 4 reminder sessions, and one cognitive bias session	✓		✓	Nearly all pigs learned the discrimination between S^- and S^+ location (33 of 36 pigs). In the test session, most pigs showed positive cognitive bias. Test for repeatability revealed no correlation between time to contact the bucket during the first(original) and second (5 weeks later) cognitive bias testing indicating a learning effect from the first test session,	(Carreras et al., 2015)
Pigs: crossbred (Landrace x Large White sows sired with Piétrain boars). Each group either consisted of Hal-free gilts, Hal-free entire males, Hal carrier gilts and Hal carrier entire males	f, m	Testing sex differences and the effects of the halothane (HAL) gene, referred to as the porcine stress syndrome gene	1	D	s; S^- bucket location with inaccessible reward; S^+ bucket location with accessible reward	s(1); bucket intermediate between S^+ and S^-	Nineteen-week-old pigs were trained individually for the CB according to the methodology described by Carreras et al. (2015): Approach bucket S^+ (rewarded with chopped apple), avoid approach S^- bucket (inaccessible chopped apple); criterion: approach S^+ faster than S- for six consecutive trials. Nine pigs did not discriminate between S+ and S- during the reminder sessions and were not tested in the cognitive bias task	After 12 training sessions and 2 reminder sessions, pigs were tested in one cognitive bias session.	✓			Neither gender differences nor effects of the HAL gene, not of their interaction were found on the latency to contact the bucket during the training and reminder sessions. The animal was classified as showing a positive, neutral, or negative cognitive bias. During cognitive bias testing, no effects of the HAL gene, of gender, and their interactions were found on latency to contact the bucket	(Carreras et al., in press)
Sheep, Romane (Lambs born from HR and LR ewes)	f, m	Prenatal chronic mild stress (aversive events such as social isolation, mixing, handling, transport, delayed feeding) in 10 highest responsive (HR) and 10 lowest responsive (LR), selected from flock of 120 ewes, during the third trimester of pregnancy. Prenatally stressed (PS) lambs were compared with control lambs	1	H	s; S^+/S^- locations on left or right side of testing arena	s(3); locations in between S^+ and S^-	Criterion learning basic discrimination task: approaching S^+ (3 companion pen mate lambs) within11s, Go-response and not approaching S^- (a blower) within 25 s (No-go response) in 2 sessions of 10 trials each.	Presentation of S^+, S^-, and ambiguous stimuli; ambiguous stimuli unrewarded	✓		✓	No differences between prenatally stressed and control lambs for S+/S- latencies, but PS lambs had longer latency to approach the ambiguous near S^- stimulus, i.e. a 'pessimistic'-like bias.	(Coulon et al., 2015)
Cattle, Holstein	m	Separation from dam	2	A	v; (white screen as S^+ vs red screen as S^-)	v(3); intermediate colors	Trained to approach screen when S^+ is shown (food reward) and not to approach screen when S^- is shown (punished with time-out). Trained to criterion: 85% correct responses for S^+ and S^- over 3 consecutive sessions	2 sessions before separation, 3 sessions after separation. 60 screens per session, with 5 trials for each of the 3 ambiguous stimuli, interspersed between S^+/S^- , ambiguous stimuli unrewarded	✓		✓	Less responses to ambiguous cues after separation, labelled pessimistic response bias	(Daros et al., 2014)

Species (strain/breed)	Sex	Experimental manipulation(s)	When	Test-arena	S^-/S^+	Ambiguous stimuli (nr.)	Training	Testing	Go/ No-go	Go/Go	Welfare	Effect(s) of experimental manipulation(s)	Reference
Cattle, Holstein	m	Hot-iron dehorning	2	A	v; (white screen as S^+ vs red screen as S^-)	v(3); intermediate colors	Trained to approach screen when S^+ is shown (food reward) and not to approach screen when S^- is shown (punished with time-out). Trained to criterion: 85% correct responses for S^+ and S^- over 3 consecutive sessions	2 sessions before separation, 3 sessions after separation. 60 screens per session, with 5 trials for each of the 3 ambiguous stimuli interspersed between S^+/S^- , ambiguous stimuli unrewarded	✓		✓	Less responses to ambiguous cues after dehorning, labelled pessimistic response bias	(Daros et al., 2014)
Sheep, Romane	f	Diazepam-induced reduction of fearfulness	2	D	s; S^+/S^- feed buckets located in opposite corners of room	s(3); intermediate positions between S^+/S^- locations	Approach bucket at S^+ (rewarded with food)/avoid approach S^- (punished by revealing blower); criterion: correct responses for two consecutive sessions of five trials	One test day, test sequence of five trials with ambiguous stimuli presented after S^+ and S^-, ambiguous stimuli unrewarded	✓			No overall effect of treatment on approaches to all stimuli, control group slower to approach near-positive ambiguous stimulus over test sessions	(Destrez et al., 2012)
Sheep, Romane	f	Daily exposure to positive events during the final four weeks of a seven-week chronic stress treatment (exposure to unpredictable and aversive events)	2	D	s; S^+/S^- feed buckets located in opposite corners of room	s (3); intermediate positions between S^+/S^- locations	Approach bucket at S^+ (rewarded with food)/avoid approach S^- (punished by revealing blower); criterion: correct responses for two consecutive sessions of five trials	One test day, test sequence of five trials with ambiguous stimuli presented after S^+ and S^-, ambiguous stimuli unrewarded	✓			Exposure to positive events induced positive bias to near-positive ambiguous stimulus (faster approach time).	(Destrez et al., 2014)
Sheep, Romane	f	Chronic stress induced by 6-week exposure to unpredictable and aversive events	1	D	s; S^+/S^- feed buckets located in opposite corners of room	s(3); intermediate positions between S^+/S^- locations	Approach bucket at S^+ (rewarded with food)/avoid approach S^- (punished by revealing blower); criterion: correct responses for two consecutive sessions of five trials	Two test days, test sequence of five trials with ambiguous stimuli presented after S^+ and S^-, ambiguous stimuli unrewarded	✓			No effect of treatment during training. Treated animals took longer to approach ambiguous stimuli during testing (negative bias) and increased approach time to near-positive stimulus over test sessions. Control animals increased approach time to near-negative ambiguous stimulus/decreased approach time to ambiguous stimulus over test sessions.	(Destrez et al., 2013)
Pigs, Large White × Landrace gilts	f	Enriched versus barren housing	1	A	a; S^- click of a dog-training clicker; S^+ note on a glockenspiel	a(1); squeak from a dog toy	Approach hatch at sound of S^+ (rewarded with apple)/avoid approach S^- (punished with plastic bag waved in the face); criterion: correct response on at least 16 out of 20 trials on one day	Five test days (test 1 after training in initial environment, test 2 and 3 after moving to opposite environment., test 4 and 5 after moving back again), two test sequences of 15 trials with ambiguous stimuli interspersed between S^+/S^-, ambiguous stimuli unrewarded	✓		✓	No effect of treatment during training. Enriched housing group more optimistic (higher nr. 'go' responses, faster approach time to ambiguous stimulus) during testing.	(Douglas et al., 2012)

Species (strain/breed)	Sex	Experimental manipulation(s)	When	Test-arena	S⁻/S⁺	Ambiguous stimuli (nr.)	Training	Testing	Go/ No-go	Go/Go	Welfare	Effect(s) of experimental manipulation(s)	Reference
Sheep, Merino	f	Restraint and isolation stress (binding legs together, no visual contact with conspecifics), 6h a day for three consecutive days	2	D	s; S^+/S^- feed buckets located in opposite corners of room	s(3) intermediate positions between S^+/S^- locations	Approach bucket at S^+ (rewarded with food)/avoid approach S^- (punished by presenting dog behind sliding panel); criterion: respond correctly for three consecutive sessions of five trials	Three test days directly after treatment, one test day 24h post-treatment, test sequence of five trials with ambiguous stimuli interspersed between S^+/S^-, ambiguous stimuli unrewarded	✓		✓	Treatment group (restraint and isolation stress) more optimistic (higher nr. of 'go' responses to all ambiguous stimuli).	(Doyle et al., 2010a)
Sheep, Merino	f	None, examining effects of repeated testing	2	D	s; S^+/S^- feed buckets located in opposite corners of room	s(9); intermediate positions between S^+/S^- locations	Approach bucket at S^+ (rewarded with food)/avoid approach S^- (punished by presenting dog behind sliding panel); criterion: respond correctly to all five trials for four out of five training days	Three test days per week for three weeks (Tue., Wed., Fri.), test sequence of five trials with ambiguous stimuli interspersed between S^+/S^-, ambiguous stimuli unrewarded	✓			Decreasing nr. of 'go' responses to middle ambiguous stimuli with repeated testing.	(Doyle et al., 2010b)
Sheep, Merino	f	Serotonin inhibitor (pCPA) treatment	2	D	s; S^+/S^- feed buckets located in opposite corners of room	s (3) intermediate positions between S^+/S^- locations	Approach bucket at S^+ (rewarded with food)/avoid approach S^- (punished by presenting dog behind sliding panel); criterion: respond correctly to all five trials for three out of four training sessions	Three test days, after three and five full days of treatment and five days post-treatment, test sequence of five trials with ambiguous stimuli presented prior to S^+/S^-, ambiguous; stimuli unrewarded	✓			Treatment group (pCPA) more pessimistic (lower number of 'go' responses to middle and near-positive ambiguous stimuli).	(Doyle et al., 2011a)
Sheep, Romane	f	Long-term exposure to unpredictable/aversive events (four weeks during training)	2	D	s; S^+/S^- feed buckets located in opposite corners of room	s(3) intermediate. positions between S^+/S^- locations	Approach bucket at S^+ (rewarded with food)/avoid approach at S^- (punished by presenting blower with paper strips attached)	Three test days after three weeks of treatment, test sequence of five trials with ambiguous stimuli presented between S^+/S^-, ambiguous stimuli unrewarded	✓		✓	Treatment group (exposed to unpredictable events) more pessimistic (lower nr. 'go' responses to near-positive ambiguous stimulus).	(Doyle et al., 2011b)
Pigs, German landrace	f	Chronic intermittent isolation paradigm (2.5 h of social isolation twice daily for three days, then once daily for four days)	2	F	s; S^+/S^- goal boxes containing food bowls located in opposite corners of room	s(3); intermediate positions between S^+/S^- locations	Approach goal box at S^+ (rewarded with food)/avoid approach goal box at S^- (unable to reach food by covering bowl with perforated plate)	Three test days post-treatment, test sequence of six trials with ambiguous stimulus at third trial, ambiguous stimuli unrewarded	✓			No effect of treatment found, all animals displayed positive bias to all ambiguous stimuli (approach latencies similar to rewarded position).	(Düpjan et al., 2013)
Rats, cLH and CNLH (bred from Sprague Dawley)	m	Strain comparison – learned helplessness (cLH) rats (animal model of depression) versus non-learned helplessness (cNLH) rats	2	9	a; S^+/S^- tones of different frequencies and sound pressure level (2 kHz, 75 dB and 9 kHz, 63 dB).	a(3); tones of intermediate frequencies (3 kHz at 72 dB, 5 kHz, 68 dB and 7 kHz, 65 dB) between S^+/S^- tones	Press lever of left wall at S^+ (rewarded with sweetened milk), press other lever at S^- (avoid punishment with foot shock); criterion: respond correctly on at least 70% of trials	Six test days, test sequence of 15 trials with ambiguous stimuli interspersed between S^+/S^-, ambiguous stimuli were unrewarded		✓		cLH rats showed a lower number of positive responses for middle and near-neg. ambiguous cues and a higher number of negative responses for the middle ambiguous cue.	(Enkel et al., 2010)

[9] Skinner box equipped with two retractable levers on opposite walls

Species (strain/breed)	Sex	Experimental manipulation(s)	When	Test-arena	S^-/S^+	Ambiguous stimuli (nr.)	Training	Testing	Go/ No-go	Go/Go	Welfare	Effect(s) of experimental manipulation(s)	Reference
Rats, cLH and CNLH (bred from Sprague Dawley)	m	Noradrenaline reuptake inhibitor and corticosterone co-treatment (neurobiological stress inducement) injected before testing	2	[9]	a; S^+/S^- tones of different frequencies and sound pressure level (2 kHz, 75 dB and 9 kHz, 63 dB).	a(3); tones of intermediate frequencies (3 kHz at 72 dB, 5 kHz, 68 dB and 7 kHz, 65 dB) between S^+/S^- tones	S^+/S^- tones of different frequencies and sound pressure level. Press lever of left wall at S^+ (rewarded with sweetened milk), press other lever at S^- (avoid punishment with foot shock); criterion: respond correctly on at least 70% of trials	Six test days, test sequence of 15 trials with ambiguous stimuli ambiguous stimuli were unrewarded		✓		Treated group ("Rbx + cort") fewer positive responses for all ambiguous cues, higher number of omissions for middle and near-pos. ambiguous cues.	(Enkel et al., 2010)
Dogs, various breeds	F, m	None, testing effects of presented stimuli with valence (picture of happy or angry human face)	2	C	v; S^- picture of angry human face; S^+ picture of happy human face	v(3) ; morphed combinations of S^+/S^-, containing 25/50/75% happy elements	Touch specific symbol (circle or triangle) at S^+ / S^- (both rewarded when chosen correctly); criterion: 12 out of 15 trials correct for both happy and angry face	Six test blocks of five trials each, with ambiguous. stimuli interspersed between S^+/S^-, ambiguous stimuli unrewarded		✓		None of the subjects learned to discriminate between S^+/S^-	(Fernandes, 2012)
Common marmosets (*Callithrix jacchus*)	f, m	Handedness of marmosets: left-handed (LH), right-handed (RH)	—	[10]	v; S^- black bowl; S^+ white bowl	v(2); near white and near black probe	Response to S^+ yielded food reward; Response to S^- yielded no reward; Criterion: 85% correct on 3 consecutive days	20 probe trials (8 S+, 8S- and 4 ambiguous, grey bowl) across 5 days; Ambiguous bowls were unrewarded; Second test series with 20 trials per ambiguous stimulus over 10 days, using the 2 ambiguous probes S^+ rewarded	✓		✓	No differences between LH and RH marmosets for inspecting the S^+ and S^-; LH treated ambiguous stimulus as negative; In second test series, LH marmosets reached for the ambiguous stimulus near S^+ slower, indicating 'pessimism'	(Gordon and Rogers, 2015)
Sheep, Lacaune	f	Unpredictable, stimulus-poor environment vs. predictable, stimulus rich environment; measurement of hemodynamic, frontal brain reactions during cognitive bias testing	2	D	s; S^+/S^- goal boxes containing food bowls located in opposite corners of room	s(3); goal boxes in between S^+ and S^-	Sheep had been used in a judgment bias experiment before. Re-training until they made a correct a Go response to the S^+ goal box (feed and salt) No-go response to the S^- goal box (straw and aversive LED light) in all 5 trials on each of 3 successive days. Enriched sheep learned faster	One session of 5 trials: S^+, S^- and three ambiguous goal box positions, randomized per sheep. In parallel, hemodynamic, frontal brain reactions were measured	✓		✓	Unexpectedly, sheep from the predictable, stimulus rich environment appeared to make more pessimistic choices. The visual assessment of the choice area in the testing apparatus lead to a general frontal cortical deactivation.	(Guldimann et al., 2015)
Rats, Lister hooded	m	Housing in unpredictable conditions (negative interventions made at random times)	2	[11]	a; S^+/S^- tones of different frequencies (2 and 4 kHz)	a(3); tones of intermediate frequencies between S^+/S^- (2.5, 3 and 3,5 kHz),	Press lever at S^+ (rewarded with food), do not press lever at S^-(avoid punishment with white noise); criterion: correct response to each tone more than 50% of the time during three sessions	10 test sessions, ambiguous stimuli interspersed between S^+/S^-, ambiguous stimuli unrewarded	✓			Animals from unpredictable housing were slower to respond to near-positive ambiguous stimulus, also tended to show fewer responses, indicating a negative bias	(Harding et al., 2004)

[10] Visual discrimination using black or white lid covering food bowl

[11] Skinner box equipped with lever

Species (strain/breed)	Sex	Experimental manipulation(s)	When	Test-arena	S-/S+	Ambiguous stimuli (nr.)	Training	Testing	Go/ No-go	Go/Go	Welfare	Effect(s) of experimental manipulation(s)	Reference
Chickens, Brown laying hens	f	Exposure to social isolation for 5 minutes in an unfamiliar environment (stressed), or left undisturbed in their group (control).	2	C	v;<br>S- white card;<br>S+ black card, or vice versa	v(3), intermediate shades of grey	Training on a two-choice task, where the left side (at presentation S+) yielded a small reward (1 mealworm), and the right side (at presentation S-) yielded a large reward (4 mealworms) or vice versa; latency to approach was recorded; criterion: 9 of 10 choices correct on two successive days of training; 20 out of 30 hens learned the task	Nine runs to S+, S- and intermediated, ambiguous stimuli (cards with intermediate shades of grey); ambiguous cues yielded no reward.		✓	✓	Response to the cue near S- and the intermediate cue was more optimistic in stressed than in control chickens; This effect was larger when the previous trial showed the S+	(Hernandez et al., 2015)
Chickens, breed unknown	m	Pharmacological reversal of isolation-induced anxiety (5 min isolation stressor) and depression (60 min isolation stressor) through treatment with clonidine and imipramine, respectively	2	A	v;<br>Innate aversive and affective stimuli used (mirror and owl image).	v(2) 75% chick/25% owl morphed silhouette and 25% chick/75% owl morphed silhouette	One trial to measure start and goal latency and farthest distance travelled in anxiety/depression condition towards S+	One trial to measure start latency and farthest distance travelled towards all stimuli after pharmacological reversal of condition	✓			Anxiety group displayed longer start latencies to ambiguous owl (near-negative) stimulus compared to control, this was not reversed by pharmacological treatment. Depression group displayed longer start latencies and shorter distance travelled towards ambiguous chick (near-positive) stimulus and ambiguous owl stimulus, this was reversed by pharmacological treatment.	(Hymel and Sufka, 2012)
Dogs of different breeds and age	f, m [12]	5 control dogs, 5 dogs with diagnosed "separation anxiety'; Dogs with separation anxiety were treated with fluoxetine in combination with a behavior modification plan starting after baseline cognitive bias testing	1, 2	F[13]	s;<br>S+/S- locations counterbalanced left/right side of the arena	s(3); locations in between S+ and S-	Training to discriminate between the S+ location (yielding food reward), and the S- location (no reward) until the adjusted speed (m.s^{-1}) to approach the S+ location exceeded the adjusted speed to approach the S-	First testing (baseline), retesting 2, 4, and 6 weeks later (separation anxiety group; controls were not tested 4 weeks after baseline). Test: Ambiguous probes interspersed between S+ and S- locations, 40 trials (9 ambiguous)	✓			Baseline measurement: Dogs with separation anxiety approached the ambiguous stimulus near S- slower than the control dogs (pessimist). Treatment normalized their behavior which became similar to that of the control dogs	(Karagiannis et al., 2015)
Grizzly bears (*Ursus arctos horribilis*)	f, m	Pilot study reward contrast - positive reward of three versus six apple slices (Experiment 1)	2	[14]	v;<br>S+/S- dark and light shade of grey presented on cardboard squares	v(3); intermediate shades of grey	Touch S+ with nose (rewarded with apple slices), touch S- with paw (rewarded with single apple slice) and vice versa; criterion: five successive training sessions (30 trials) at 90% accuracy	Four test sessions, test sequence of 30 trials with one ambiguous stimulus interjected between S+/S- once every 10 trials, ambiguous stimuli rewarded with secondary reinforcer (clicker)		✓		No difference found between groups receiving three or six slices as high reward, no bias in response toward ambiguous stimuli found.	(Keen et al., 2014)

[12] Some neutered

[13] Similar to F with respect to the start and goal positions; distances varied, and the tests were not performed in an enclosed arena, but at the owner's home

[14] Stimuli were presented outside animal enclosure, with animals responding through the fence

Species (strain/breed)	Sex	Experimental manipulation(s)	When	Test-arena	S^-/S^+	Ambiguous stimuli (nr.)	Training	Testing	Go/ No-go	Go/Go	Welfare	Effect(s) of experimental manipula-tion(s)	Reference
Grizzly bears (*Ursus arctos horribilis*)	f, m	Enrichment session pre-testing with three types of enrichment item (low, medium and high interest item) (Experiment 2)	2	[14]	v; S^+/S^- dark and light shade of grey presented on card–board squares	v(3); inter-mediate shades of grey	Touch S^+ with nose (rewarded with four apple slices), touch S^- with paw (rewarded with single apple slice) and vice versa; criterion: five successive training sessions (30 trials) at 90% accuracy	18 test sessions, test sequence of 30 trials with one ambiguous stimulus interjected between S^+/S^- once every 10 trials, ambiguous stimuli rewarded with secondary reinforcer (clicker)		✓	✓	No effect found of enrichment condition, no bias in response toward ambiguous stimuli found.	(Keen et al., 2014)
Dogs, different breeds and age	f, m[12]	Responses to ambiguous stimuli in the cognitive bias task after intranasal administration of oxytocin or placebo, in a com-municative or non-communicative condition	2	F[15]	s; S^+/S^- left or right side of the test arena	s(1) interme-diate be-tween S+ and S-	Experimenter stood between S^+ (bowl with food reward) and S^- (empty bowl), experimenter called dog with name + "look", and put the food bowl on the floor; owner released dog to start trial. Criterion: latency to reach negative location longer than reaching positive location (calculated by Wilcoxon test).	Re-training on S+, S in the presence (communicative context) or absence (non-communicative condition) of the experimenter, followed by testing response to the ambig-uous food bowl location , (order of trials: negative, positive, ambiguous trial) in the pres-ence or absence of the experi-menter		✓		Oxytocin-treated dogs had higher positive expectancy than control dog when presented the ambiguous bowl position; the communicative condition (experi-menter present) increased the positive expectancy	(Kis et al., 2015)
Mice, C57BL/6N	f	Exp. 1: Validating assessment of differing behavioral outcomes in response to an ambiguous stimulus between mice anticipat-ing a positive or negative event (exp. 1)	—	E	s; S^- air-puff upon reaching hole at end of maze arm; S^+ hole at end of maze arm was exit of maze	s(1); maze arm in between S^+ and S^- arms	Optimistically trained mice could use either of 2 maze arms to exit maze, pessimistically trained mice received air-puff upon reaching a hole at the end of either of 2 available maze arms, 13 trials over 3 training days	Response to unfamiliar maze arm, located in between S^+/S^- arms, single testing trial per mouse	✓			Optimistically trained mice had de-creased latencies to reach the hole in the unfamiliar maze arm	(Kloke et al., 2014)
Mice, C57BL/6J	f	Exp. 2: Validating applicability of a spatial location as a discrimi-natory stimulus for mice (exp. 2)	—	E	s; S^- air-puff upon reaching hole at end of maze arm; S^+ hole at end of maze arm was exit of maze	s(3); maze arms in between S^+ and S^- arms	Mice were trained to discriminate between positive (exit) and negative (air-puff) arm on either side of maze, 21 trials over 4 training days, criterion: shorter latency to reach positive arm than negative arm	Response to ambiguous arms (unfamiliar central arm, near-negative arm or near-positive arm), single testing trial per mouse	✓			Mice were faster to reach the near-positive arm and slower to reach the hole in the near-negative arm	(Kloke et al., 2014)
Mice, 5-HTT +/+, +/- and -/- (C57Bl/6J background)	f	Pilot study with 5-HTT knockout mice (-/- with anxiety- and depression-like phenotype)	1	E	s; S^- air-puff upon reaching hole at end of maze arm; S^+ hole at end of maze arm was exit of maze	s(1); maze arm in between S^+ and S^- arms	Mice were trained to discriminate between positive (exit) and negative (air-puff) arm on either side of maze, 25 trials over 5 training days, criterion: shorter latency to reach positive arm than negative arm	Response to unfamiliar maze arm, located in between S^+/S^- arms, single testing trial per mouse	✓			Non-significant trend for 5-HTT -/- mice to have highest latency and +/+ mice to have lowest latency to reach the hole in the central arm	(Kloke et al., 2014)

[15] Similar to F with respect to the start and goal positions; the tests were not performed in an arena, and only one ambiguous location (intermediate between S^+ and S^-) was presented during testing

Species (strain/breed)	Sex	Experimental manipulation(s)	When	Test-arena	S^-/S^+	Ambiguous stimuli (nr.)	Training	Testing	Go/ No-go	Go/Go	Welfare	Effect(s) of experimental manipula-tion(s)	Reference
Rats, Sprague Dawley	m	Single i.p injections of dopamin-ergic precursor L-DOPA (2, 4, 8 $mg.kg^{-1}$), D2 receptor antagonist haloperidol (0.01, 0.02, 0.05 $mg.kg^{-1}$), or serotonin reuptake inhibitor escitalopram (0.5, 1, 2 $mg.kg^{-1}$) (randomized Latin square design with saline as vehicle control); one-week wash-out intervals between doses (Experiment 1)	2	[16]	a; S^- 9000 Hz tone: right lever to avoid delayed foot-shock; S^+ 2000 Hz tone: left lever to gain sucrose reward, or vice versa.	a(1); 5000 Hz tone)	Training on a operant task with one auditory stimulus that predicts reward, the other that predicts punishment. Introduction of an intermediate ambiguous stimulus that lead to no consequences. After separate e training on S^- and S^+: pseudo-random presentation of S^- and S^+ (20:20). Training to criterion of 70% correct discrimination performance.	Pseudorandom presentation of S^-, S^+, and ambiguous stimulus (20:20:10) during single testing session per dose		✓		No effect: L-DOPA, escitalopram;<br>Effect: haloperidol deceased positive and negative lever presses to the ambiguous tone; also increased omissions were measured	(Kregiel et al., 2016a)
Rats, Sprague Dawley	m	daily injection of L-DOPA (8 $mg.kg^{-1}$), haloperidol (0.05 $mg.kg^{-1}$), escitalopram (2 $mg.kg^{-1}$) for 2 weeks; the trypto-phan hydroxylase inhibitor 4-cloro-DL-phenylalanine methyl ester (PCPA, 400 $mg.kg^{-1}$) was administered daily once on the first two days of the first and second week (Experiment 2)	2	[16]	a; S^- 9000 Hz tone: right lever to avoid delayed foot-shock; S^+ 2000 Hz tone: left lever to gain sucrose reward, or vice versa.	a(1); 5000 Hz tone)	Trained rats from exp. 1 were re-used two weeks after the end of exp. 1; they were assigned random-ly to five groups (4 groups received a drug, one group served as saline control)	After chronic administration of test compounds (or saline) the animals were tested in one session		✓		No effect: haloperidol, escitalopram, PCPA;<br>Effect: L-DOPA increased positive lever presses in response to ambiguous tone	(Kregiel et al., 2016a)
Rats, Sprague Dawley	m	Treatment with the irreversible anandamide hydrolysis inhibitor URB597 in three doses (experiment 1)	2	[16]	a; S^- 9000 Hz tone: right lever to avoid delayed foot-shock S^+ 2000 Hz tone: left lever to gain sucrose reward, or vice versa.	a(1); 5000 Hz tone	Training on a operant task with one auditory stimulus that predicts reward, the other predicts punish-ment. Introduction of an intermedi-ate ambiguous stimulus that lead to no consequences. After separate e training on S^- and S^+: pseudo-random presentation of S^- and S^+ (20:20). Training to criterion of 70% correct discrimination performance.	Pseudorandom presentation of S^-, S^+, and ambiguous stimulus (20:20:10) during single testing session, in baseline session and 30 or 60 minutes after drug administration		✓		Experiment 1: URB597 increased "opti-mistic" choices to the ambiguous tone at the highest dose tested (1 mg.kg-1).	(Kregiel et al., 2016b)

[16] Two-lever Skinnerbox with sucrose reward and footshock punishment

Species (strain/breed)	Sex	Experimental manipulation(s)	When	Test-arena	S^-/S^+	Ambiguous stimuli (nr.)	Training	Testing	Go/ No-go	Go/Go	Welfare	Effect(s) of experimental manipula-tion(s)	Reference
Rats, Sprague Dawley	m	Treatment with URB597. the cannabinoid receptor type 1 (CB1) inverse agonist AM251, the CB2 inverse agonist AM630, combination URB597-AM251, and combination URB597-AM630 (Experiment 2)	2	[16]	a; S^- 9000 Hz tone: right lever to avoid delayed foot-shock S^+ 2000 Hz tone: left lever to gain sucrose reward, or vice versa.	a(1); 5000 Hz tone	Training on a operant task with one auditory stimulus that predicts reward, the other predicts punish-ment. Introduction of an intermedi-ate ambiguous stimulus that lead to no consequences. After separate e training on S^- and S^+: pseudo-random presentation of S^- and S^+ (20:20). Training to criterion of 70% correct discrimination performance.	Pseudorandom presentation of S^-, S^+, and ambiguous stimulus (20:20:10) during single testing session, in baseline session and 30 or 60 minutes after drug administration		✓		Experiment 2:AM251 and AM630 alone had no effect, but both antagonized the effect of URB597 (see result experiment 1)	(Kregiel et al., 2016b)
Chickens, domestic breed	f	Housing in battery cage until 67 weeks of age; testing 2 and 4 months after rehousing to ground housing with litter, laying nest and perch	1	F	s; S^- empty bowl; S^+ bowl contain-ing the food reward	s(3); bowl in between S^+ and S^- location	Training until approach to bowl with food in less than 2 min for 3 con-secutive trials Eight trials per training session for 12 or more sessions	3 runs with ambiguous probe positions mixed with 10 runs with unambiguous positions at 2 months after rehousing. This was repeated at 4 months after rehousing.	✓		✓	Hens took longer to reach center ambig-uous position four months after rehousing compared to two months after rehousing. No differences at other positions.	(Lindström, 2010)
European starling (*Sturnus vulgaris*)	f, m	Housing in enriched or unen-riched cages	2	A	d (visuotem-poral); S^-: light on 2 s; S^+ light on 10 s	d(8) interme-diate dura-tion stimuli	Training to peck one colored key for immediate reward (after S^+) and a different colored key for a delayed reward (after S^-). Maximum 54 trials or 2,5 h per session. Criterion: 65% correct for 3 consecutive sessions.	360 ambiguous probe trials (light on durations in between S^+ and S^-) across 10 days, each session had 18 reinforced trials and 36 probe trials		✓	✓	Subset of animals was significantly more likely to choose the S^+ associated key when housed in enriched environment; overall no effects of enrichment were found.	(Matheson et al., 2008)
Rats, Sprague-Dawley	m	Subcutaneous treatment with 0.001 $mg.kg^{-1}$ oxytocin, 5 minutes before testing trials	2	[17]	d (Visuospatial); S^- white com-partment with smooth floor; S^+ black com-partment with textured floor, and vice versa	d (2) Visuospatial) ; white compartment with textured floor and black com-partment with smooth floor	After habituation to the place preference apparatus, rats received 2 to 5 training sessions with 6 aversive trials (compartment with quinine soaked food) and 6 reward-ed trials (compartment with palata-ble food), presented in a random-ized order. Criterion of learning was that latency to approach food bowl in rewarded compartment was at least 5 seconds shorter that ap-proach to aversive compartment	One testing session on each of two successive days. Ambigu-ous trials were not rewarded, whereas the food bowls con-tained food as during training in the S^- and S^+ trials				Oxytocin treatment had no effect on the latency to approach the food bowl during ambiguous trials. However, rats behaved in ambiguous trials with short latencies, suggesting that they showed a positive cognitive bias. The dose of oxytocin administered may have been ineffective.	(McGuire et al., 2015)

[17] Modified conditioned place preference apparatus (grey start box, with a larger black and a larger white box on either side of the start box)

Species (strain/breed)	Sex	Experimental manipulation(s)	When	Test-arena	S^-/S^+	Ambiguous stimuli (nr.)	Training	Testing	Go/ No-go	Go/Go	Welfare	Effect(s) of experimental manipulation(s)	Reference
Dogs various breeds)	f, m	Animals which perform destructive separation-related behavior	1	G	s;<br>S^- bowl on one side;<br>S^+ bowl on the other side of the arena	s(3); bowl in between S^- and S^+ location	Training to approach the S+ (bowl containing food) and to suppress approaching the S- (empty bowl), for at least 15 trials.; S+ and S- were presented in pseudorandom order. Learning criterion: when from the preceding three positive trials and negative trials, the longest latency to reach the S+ was shorter than any of the latencies to reach the S-	3 probe trials, ambiguous locations between S+ and S-. Each probe separated by 4 trials with S^-/S^+. Measure: latency to approach bowl.	✓		✓	Dogs with higher destructive separation-related behavior showed higher latencies at mid position, interpreted as more pessimistic response.	(Mendl et al., 2010)
Dogs, various breeds	f, m	Separation from owner vs owner present (within subjects design)	2	G	s;<br>S^- bowl on one side;<br>S^+ bowl on the other side of the arena	s(3); bowl in between S^- and S^+ location	Blocks of 10 trials, with 5 S^- (empty bowl) and 5 S^+ (bowl containing food reward Criterion: statistically significant difference in latency to approach S^- and S^+ or maximum of 120 trials	Two blocks of 26 trials: per block 6 ambiguous probe trials interspersed with 20 standard trials	✓		✓	No effect of separation from owner on cognitive bias.	(Müller et al., 2012)
Pigs (Duroc x Yorkshire and Duroc x Danish Landrace versus Göttingen minipig)	f	Genetic background and restraint (1-5 minutes)	1, 2	C	a;<br>S^+ S^-<br>2 tones (either 200 or 1000Hz	a (3); intermediate tones)	Trained to perform operant response in one location following S^+, and another location following S^- Trained to criterion of three consecutive sessions with at least 4 out of 5 correct choices for both S^+ and S^-.	Two phases<br>Phase 1 testing: four sessions of 16 trials, of which 3 forced and 10 free trials with S^+ and S^-, and 3 trials with ambiguous tones.<br>Phase 2: isolation for 5 minutes before and 15 minutes after testing, testing as in Phase 1.		✓	✓	No effect of breed or isolation on judgment bias.	(Murphy et al., 2013)
Dairy calves (Holstein)	m	Hot iron de-horning	2	A	v;<br>S^-<br>red video screen;<br>S^+<br>white screen	v(3 intermediate colors between red and white	Trained to nose touch a screen when S^+ is shown to receive milk and not to touch the screen when S^- is shown to prevent a time out. Trained to criterion: 90% correct responses over 3 consecutive sessions in experiment1, 85% correct responses over 3 consecutive sessions in experiment 2.	3 sessions before disbudding, 2 sessions after disbudding. 60 screens per session, with 8% of trials for each of the 3 ambiguous stimuli.	✓		✓	Fewer responses to the intermediate and near-negative stimuli after de-horning.	(Neave et al., 2013)

Species (strain/breed)	Sex	Experimental manipulation(s)	When	Test-arena	S^-/S^+	Ambiguous stimuli (nr.)	Training	Testing	Go/ No-go	Go/Go	Welfare	Effect(s) of experimental manipulation(s)	Reference
White-lipped peccary (*Tayassu pecari*)	?	Trapping before the second judgment bias test	2	[18]	a;<br>S^- horn;<br>S^+ whistle	a(1) bell	Go response to S^+, i.e. approach to a food dispenser; No-go response to S^-, staying away from food dispenser (cut-off: 60 seconds)	30 trials per test over 2 days, 10 ambiguous trials (bell, unrewarded). Animals were exposed to three tests one basic (T1), 7 days later T2 (30 minutes after trapping) and T3 (7 days after T2) speed (m.s^{-1}) to get to the feed dispenser was registered.	✓		✓	The peccary discriminated between S^+ and S^-; Whereas proportion of go-responses was intermediate between S^+ and S^- in testing periods 1 and 3 (no trapping), it was near the S^- after trapping in testing period 2, suggesting a pessimistic judgment of this cues	(Nogueira et al., 2015)
Mice, CD1	f	Daily handling with two different methods, tail and cup (week 3-18), home cage recording (week 19-25). Judgment bias training and test in week 26 & 27, animals were also handled in training and testing weeks.	1,2	E[19]	s	S(4)	Running towards the positive arms (S^+) turns off the overhead lights (400 lux) and delivers a food pellet. Running towards the negative arms (S^-) turns on overhead lights and white noise. 6 days of training were performed, no learning criterion, but data shows increased discrimination between positive and negative arms	One test session of one minute in which all arms were open. The four arms in between the S^+ and S^- were the ambiguous cues. Exploration time of each arm was examined	✓			The different handling methods had no effects on duration and frequency of exploration of ambiguous arms in the test. Test shows potential since mice explored near positive arms more than the near-negative arms.	(Novak et al., 2015)
Mice, CD-1 and C57BL/6/JRcc	f	Unpredictable chronic mild stress (UCMS): after reaching criterion during the training phase, all mice underwent unpredictable chronic mild stress during a 3-week period. During stress treatment, mice were trained on a partial reinforcement schedule, i.e. a proportion of the trials was unrewarded. Half of the mice served as controls and were not subjected to UCMS.	2	I	t;<br>S^+/S^- coarse or fine sandpaper associate the a high or low value reward	t(3) intermediate grades of sand paper)	Training positive trials: compartment and goal pot covered with fine or coarse sandpaper with a hidden almond flake, a high value reward vs. compartment and goal pot without sandpaper (i.e. incorrect choice);<br>negative trial: compartment and goal pot without sandpaper with a hidden oat flake, a low value reward, vs. compartment with the other grade of sandpaper (i.e. the incorrect choice). Criterion: series of 10 correct choices in a series of 14 trials.	Three judgment bias sessions with 15 trials each (six positive, six negative and 3 intermediate stimulus presentations); optimistic choices were trials in which the mice dig in the goal pot and compartment covered with sandpaper		✓	✓	Non-learners (2 CD-1, 4 C57BL mice) were excluded. Overall, CD-1 mice were faster learners.<br>UCMS tended to decrease responding to the positive (almond) trials.<br>The control mice of both made a graded response to the intermediate (ambiguous) cues, and made more optimistic responses to the near positive cue, and less optimistic responses to the near negative cue, whereas the UCMS mice made similar responses to all intermediate cues. Also, UCMS responded faster to the intermediate cues.	(Novak et al., 2016)
Rats, Sprague Dawley	f	Social stress (resident-intruder paradigm)	2	C	a;<br>S^+/S^- two tones of 9000 or 2000 Hz	a(1); intermediate tone of 5000 Hz	Trained to press a lever at S^+ to gain sucrose solution and to press a second lever at S^- to avoid a shock. Trained to criterion of 70% correct responses for each lever for 3 consecutive sessions.	One session of 20 S^+, 20 S^- and 10 ambiguous stimuli before social stress, and one session of 20 S^+, 20 S^- and 10 ambiguous stimuli after social stress.		✓		More responses on S^- lever after social stress (pessimistic response).	(Papciak et al., 2013)

[18] Part of the enclosure where the peccary were kept

[19] The whole radial arm maze was used

Species (strain/breed)	Sex	Experimental manipulation(s)	When	Test-arena	S^-/S^+	Ambiguous stimuli (nr.)	Training	Testing	Go/ No-go	Go/Go	Welfare	Effect(s) of experimental manipulation(s)	Reference
Rats, Lister hooded	m	Unpredictable housing (negative interventions made at random times)	2	[20]	a; S^+/S^- : two tones with different frequencies(either 2 or 4 kHz), counter-balanced	a(14); single-frequency tones (1.6-4.4 kHz with 200 Hz increments) or dual-frequency tone consisting of combined S^+ and S^-	Trained to press a lever at S^+ to gain 2 food pellets and to press a second lever at S^- to gain 1 food pellet. Criterion: 3 consecutive sessions of performance which was significantly greater than chance-level for each trial type.	6 single-frequency tone testing sessions (3 before and 3 after treatment/156 trials or 60 minutes with 50% S) and 2 dual-frequency tone sessions (1 before and 1 after treatment/64 trials or 30 minutes with 50% S), ambiguous trials were not rewarded.		✓	✓	Control group decreased responses to ambiguous tones over time, treated group did not. No other differences between groups.	(Parker et al., 2014)
Capuchin monkeys *(Cebus apella)*	f, m	None, correlation of judgement bias with stereotypical behaviors	1	C	v; S^+/S^- : large or small striped panel, counter-balanced	v(1); striped panel of intermediate size	Monkeys were trained to respond to S^+ by retrieving preferred reward from one location, and S^- by retrieving non-preferred reward from another location	Five 20-trial sessions on consecutive days with 9 S^+, 9 S^-, and 2 ambiguous trials per session.		✓	✓	Negative correlation between probability to choose the positive reward and amount of stereotypy displayed.	(Pomerantz et al., 2012)
Sprague Dawley rats	m	None, classification of rats as optimistic or pessimistic before testing them in a rat slot machine task	1	C	a; S+/S- two tones of 9000 or 2000 Hz	a(1); intermediate tone of 5000 Hz	The rats were trained to press one lever when a 'positive' tone (2000 Hz at 75 dB) signaled a reward (5% sucrose solution) and to press second lever when another, 'negative' tone (9000 Hz at 75 dB) signaled punishment (0.5 mA foot shock, duration: 10 s). Criterion: 70% correct responses on each lever, over three consecutive discrimination sessions	cognitive judgment bias as a trait was assessed across a series of 10 consecutive tests at one-week intervals. Based on the average cognitive bias index obtained across the 10 tests, rats were classified as optimistic or pessimistic		✓		No experimental manipulations	(Rafa et al., 2016)
Rats, 71st and 72nd generation of selection (cLH and CNLH) lines originating from Sprague-Dawley	m	Selection lines for high or low learned helplessness tested before and after 4 weeks of environmental enrichment	1, 2	E	s; 2 arms at opposite location in a radial arm maze	s(3); arms at intermediate positions	Training to retrieve "fruit loops" cereal (US^+) or avoid "fruit loops" cereal soaked in quinine (US^-) from reference locations. 12 trials per session, 6 with US^+ and 6 with US^-, of which 1 trial had no reward at S^+ location (partial reinforcement). Rats were trained to individual criterion: significant difference in latencies to approach the S^+ and S^- goal pots on two consecutive days (Mann–Whitney U Test, one-tailed, $p \leq .05$). Training for a minimum of 3 days but for no more than 7 days.	3 testing sessions with 13 trials per session, of which 3 ambiguous (1 trial per ambiguous location per session, totaling 3 trials per ambiguous location).	✓			Higher latencies to reach all three ambiguous goal-pots in selection line for high learned helplessness compared to low learned helplessness line (more negative bias); both groups showed reduced latency time to dip nose into any goal pot following enriched housing.	(Richter et al., 2012)

[20] Retractable lever on either side of the food trough

Species (strain/breed)	Sex	Experimental manipulation(s)	When	Test-arena	S-/S+	Ambiguous stimuli (nr.)	Training	Testing	Go/ No-go	Go/Go	Welfare	Effect(s) of experimental manipulation(s)	Reference
Rats, Sprague–Dawley	m	Selection of rats, based on 1 weekly session of cognitive bias testing over 10 weeks as 'optimistic' or ' pessimistic' (baseline). Then, half of the optimistic and half of the pessimistic rats received daily1-h immobilization sessions over a period of 3 weeks, whereas the other half (controls) was handled. Effects of immobilization stress were tested once per week during this period in the judgement bias task	1, 2	16	a;<br>S^- 9000 Hz tone: right lever to avoid delayed foot–shock;<br>S^- 2000 Hz tone: left lever to gain sucrose reward, or vice versa.	a(1); 5000 Hz tone	Training on a operant task: S^+ auditory stimulus predicted sucrose reward, the other S^- auditory stimulus predicted food shock, avoidance could be achieved when right lever was pressed. . After separate e training on S^- and S^+: pseudo-random presentation of S^- and S^+ (20:20). Training to criterion of 70% correct discrimination performance.	Pseudorandom presentation of S^-, S^+, and ambiguous stimuli (20:20:10). ambiguous stimulus lead to no consequences.		✓		The stability of the cognitive bias response during baseline measurement in the course of 10 weeks suggests that 'pessimism' and ' optimism' are behavioral traits. Rats that underwent repeated immobilization stress of both the 'optimistic' and the 'pessimistic' group (according to baseline testing) were more pessimistic than the handled control rats, compared with their baseline values. The two control groups did not change their bias during the immobilization period.	(Rygula et al., 2013)
Rats, Sprague–Dawley	m	Acute pharmacological stimulation of the serotonin (5-HT), noradrenaline (NA) and dopamine (DA) systems<br>5HT: selective serotonin reuptake inhibitor (SSRI) citalopram (1, 5 and 10 $mg.kg^{-1}$);<br>NA: noradrenaline reuptake inhibitor desipramine (1, 2 and 5 $mg.kg^{-1}$);<br>DA: DA (and to a lesser extent NA and 5-HT) releaser d-amphetamine (0.1, 0.5 and 1 $mg.kg^{-1}$)	2	16	a;<br>S^- 9000 Hz tone: right lever to avoid (delayed) foot–shock;<br>S^+ 2000 Hz tone: left lever to gain sucrose reward, or vice versa	a(1); 5000 Hz tone	Training on a operant task: S^+ auditory stimulus predicted sucrose reward when pressing the left lever, the other S^- auditory stimulus predicted food shock, avoidance could be achieved when right lever was pressed. After separate training on S^- and S^+: pseudo-random presentation of S^- and S^+ (20:20). Training to criterion of 70% correct discrimination performance.	Pseudorandom presentation of S^-, S^+, and ambiguous stimulus (20:20:10) during single testing session		✓		5-HT stimulation induced negative cognitive bias at 1 $mg.kg^{-1}$ citalopram (reduction of optimistic lever presses). At higher dosages (5 or 10 $mg.kg^{-1}$) positive cognitive bias was induced by reducing pessimistic lever presses. NA stimulation induced negative bias in all tested doses of desipramine by reducing optimistic lever presses and increasing pessimistic lever presses. DA stimulation at 1 $mg.kg^{-1}$ d-amphetamine induced positive bias by reducing pessimistic lever presses. No effects were found at lower doses (0.1 and 0.5 $mg.kg^{-1}$).	(Rygula et al., 2014a)
Rats, Sprague–Dawley	m	Chronic, daily administration of psychostimulants (amphetamine or cocaine) for a duration of 2 weeks	2	16	a;<br>S^- 9000 Hz tone: right lever to avoid (delayed) foot–shock;<br>S^+ 2000 Hz tone: left lever to gain sucrose reward, or vice versa	a(1); 5000 Hz tone	Training on a operant task: S^+ auditory stimulus predicted sucrose reward when pressing the left lever, the other S^- auditory stimulus predicted food shock, avoidance could be achieved when right lever was pressed. After separate training on S^- and S^+: pseudo-random presentation of S^- and S^+ (20:20). Training to criterion of 70% correct discrimination performance.	Pseudorandom presentation of S^-, S^+, and ambiguous stimuli (20:20:10) during single testing session		✓		Treatment impaired both groups of rats in their ability to discriminate between and/or react to the S^+/S^-. neither drug resulted in a significant effect on the interpretation of the ambiguous stimulus.	(Rygula et al., 2015c)

Species (strain/breed)	Sex	Experimental manipulation(s)	When	Test-arena	S^-/S^+	Ambiguous stimuli (nr.)	Training	Testing	Go/ No-go	Go/Go	Welfare	Effect(s) of experimental manipulation(s)	Reference
Rats, Sprague–Dawley	m	Acute treatment with valproic acid (100, 200, 400 $mg.kg^{-1}$), or lithium chloride (10, 50, 100 $mg.kg^{-1}$) (Latin square design), with 1-week washout period between drug administrations	2	16	a; S^- 9000 Hz tone: right lever to avoid (delayed) foot-shock; S^+ 2000 Hz tone: left lever to gain sucrose reward, or vice versa	a(1); 5000 Hz tone	Training on a operant task: S^+ auditory stimulus predicted sucrose reward when pressing the left lever, the other S^- auditory stimulus predicted food shock, avoidance could be achieved when right lever was pressed. After separate training on S^- and S^+: pseudo-random presentation of S^- and S^+ (20:20). Training to criterion of 70% correct discrimination performance over three consecutive days.	Pseudorandom presentation of S^-, S^+, and ambiguous stimuli (20:20:10) during single testing session.		✓		Valproic acid had no effects in any dose tested; lithium at the dose of 50, but not 10 and 100 $mg.kg^{-1}$ affected responding to the ambiguous tone cue, indicating an optimistic bias.	(Rygula et al., 2015a)
Rats, Sprague–Dawley	m	Tickling of the rats, vs. handling without tickling in a cross over schedule (within subjects comparisons). Subdivision of rats into "laughing when tickled" (emission of 50-kHz ultrasonic vocalizations) and "not laughing when tickled" group.	2	16	a; S^- 9000 Hz tone: right lever to avoid delayed foot–shock; S^- 2000 Hz tone: left lever to gain sucrose reward, or vice versa.	a(1); 5000 Hz tone.	Training on a operant task: S^+ auditory stimulus predicted sucrose reward when pressing the left lever, the other S^- auditory stimulus predicted food shock, avoidance could be achieved when right lever was pressed. After separate training on S^- and S^+: pseudo-random presentation of S^- and S^+ (20:20). Training to criterion of 70% correct discrimination performance.	Pseudorandom presentation of S^-, S^+, and ambiguous stimuli (20:20:10) during single testing session.		✓	✓	The laughing when tickled rats showed a positive bias toward the ambiguous tone; no effects of tickling were seen toward the positive and negative tones. Both subgroups showed slightly more response omissions to ambiguous tone	(Rygula et al., 2012)
Rats, Sprague–Dawley	m	Acute pharmacological stimulation of the dopamine (DA) system by administration of either cocaine or mazindol	2	16	a; S^- 9000 Hz tone: right lever to avoid (delayed) foot–shock; S^+ 2000 Hz tone: left lever to gain sucrose reward	a(1); 5000 Hz tone	Training on a operant task: S^+ auditory stimulus predicted sucrose reward when pressing the left lever. the other S^- auditory stimulus predicted food shock, avoidance could be achieved when right lever was pressed. After separate training on S^- and S^+: pseudo-random presentation of S^- and S^+ (20:20). Training to criterion of 70% correct discrimination performance.	Pseudorandom presentation of S^-, S^+, and ambiguous stimuli (20:20:10) during single testing session		✓		Cocaine had no effect on the rats' ambiguous cue interpretation. Administration of mazindol resulted in a negative bias by reducing optimistic lever presses and increasing pessimistic lever presses.	(Rygula et al., 2014b)
Rats, Sprague–Dawley	m	Selection of "optimistic" and "pessimistic" rats, based on responding in Judgment bias task. Then: assessment of motivation to gain food reward and to avoid punishment using a progressive ration of reinforcement schedule	—	16	a; S^- 9000 Hz tone: right lever to avoid (delayed) foot–shock; S^+ 2000 Hz tone: left lever to gain sucrose reward	a(1); 5000 Hz tone	Training on a operant task: S^+ auditory stimulus predicted sucrose reward when pressing the left lever. the other S^- auditory stimulus predicted food shock, avoidance could be achieved when right lever was pressed. After separate training on S^- and S^+: pseudo-random presentation of S^- and S^+ (20:20). Training to criterion of 70% correct discrimination performance.	Pseudorandom presentation of S^-, S^+, and ambiguous stimuli (20:20:10) during single testing session; rats were selected for optimistic and pessimistic traits, motivation for food and avoidance of punishment was investigated.		✓		The two groups did not differ for avoidance of punishment; the optimistic group showed a higher motivation to gain food reward.	(Rygula et al., 2015b)

Species (strain/breed)	Sex	Experimental manipulation(s)	When	Test-arena	S^-/S^+	Ambiguous stimuli (nr.)	Training	Testing	Go/ No-go	Go/Go	Welfare	Effect(s) of experimental manipulation(s)	Reference
Chickens (*Gallus gallus*)	f	Testing twice (at 4 and 5 days of age), experiment 1	2	A	v; innate aversive and affective stimuli used (mirror and owl image).	v(2) 75% chick/25% owl morphed silhouette and 25% chick/75% owl morphed silhouette	One trial to measure the latencies to leave the start box and reach the goal box (S^+ mirror image of the chick) on day 4	One trial to measure the latencies to leave the start box and reach the goal box (S^-: image of owl, S^+: image of chick, ambiguous cues: three morphs between chick and owl) on day 5		✓		Latency to reach end of runway increased with degree of similarity of stimuli with owl silhouette.	(Salmeto et al., 2011)
Chickens (*Gallus gallus*)	f	3 groups of chickens: 5 minutes isolation, 60 minutes isolation, or no isolation (control), experiment 2	2	A	v; S^- mirror image of chick tested; S^+ owl silhouette	v(3); morphs between chick and owl.	One trial to measure the latencies to leave the start box and reach the goal box (S^+ mirror image of the chick) on day 4	One trial to measure the latencies to leave the start box and reach the goal box (S^-: image of owl, S^+: mirror image of chick, ambiguous cues: three morphs between chick and owl) on day 5 or 6		✓		Latency to reach end of runway increased with degree of similarity of stimuli with owl silhouette.60-minutes of isolation increased latencies more than 5 minutes isolation. (3-minutes isolation interpreted as anxiety-like phenotype, 60-minutes isolation interpreted as depression-like phenotype)	(Salmeto et al., 2011)
Sheep, Merino ewes	f	Sheering, vs. unshorn controls; Sheep were shorn immediately before entering the cognitive bias facility	2	D	s; S^- bucket on one side of the arena); S^+ bucket on the other side	s(3); buckets in between S^- and S^+ location	Discrimination between location of S^- (dog exposed behind sliding panel) and S^+ (bucket containing the food reward); criterion: no approach of S^- bucket on 3 of 4 successive days	Sheep were tested in two cohorts, each consisting of 3 shorn sheep and 3 unshorn controls	✓		✓	Shorn sheep of cohort 1 showed a positive bias; they approached the ambiguous bucket locations more than the unshorn sheep did. This effect was absent in cohort 2. Release from sheering appeared to be judged as positive. Sheering-induced stress was confirmed by increase plasma cortisol levels and decreased eosinophil count.	(Sanger et al., 2011)
Pigs, crossbred Large White X Landrace	f, m	Housing at two different stocking densities: low (conventional) vs. higher space allowance	1	F	s; S^- bucket on one side of the arena); S^+ bucket on the other side	s(3); buckets in between S^- and S^+ location	Discrimination between location of S^- (empty bucket) and S^+ (bucket containing pelleted weaner food); Criterion: statistically significant difference in latency to approach S^-, S^+	3 test days (each preceded by a day with the bucket in in the trained S^-, S^+ position) on test days, the bucket was located in a pseudorandom sequence in S^+, S^- and each of the three ambiguous locations.		✓	✓	No effects of space allowance on learning the discrimination between $S^-$and S^+. No effects on the latencies to approach the originally trained and the ambiguous bucket positions. No effects on physiological measures (salivary cortisol, α-amylase, but more sitting behavior and more skin lesions in pigs with low space allowance	(Scollo et al., 2014)
Chickens (*Gallus gallus*)	f	Chicks were housed in groups of 8 in round pens divided in three-area's: dark area, litter area (floor covered with saw dust), feed area from hatching until the end of cognitive bias testing at 8 weeks of age. Each of the three area's was shut off for four days in following weeks.	1	F	s; S^- bucket on one side of the arena); S^+ bucket on the other side	s(3); bowl in between S^- and S^+ location	Discrimination between location of S^- (bowl with a piece of puffed rice soaked in quinine sulphate solution) and S^+(mealworm); Criterion: 2 s mean difference in running speed to approach the S^-, S^+ location	3 test series (with each treatment) in 3 successive week, each starting with the presentation of the bowl at S^- and S^+ position; The bowl was then located in S^+, S^- and each of the three ambiguous locations.		✓	✓	Chicks appeared to continue learning during the 3 testing weeks. Shorter running speed to the near negative bowl position after shutting off the litter area may indicate that this manipulation affected the chicks les negatively than shutting off each of the other two areas.	(Seehuus et al., 2013)

Species (strain/breed)	Sex	Experimental manipulation(s)	When	Test-arena	S^-/S^+	Ambiguous stimuli (nr.)	Training	Testing	Go/ No-go	Go/Go	Welfare	Effect(s) of experimental manipula-tion(s)	Reference
Dogs, various breeds	f, m	Different breeds and ages. Individual differences between dogs.	1	[21]	a;<br>S^- high or low tone;<br>S^+ the other of the two tones,	a (9); tones between the ones associ-ated with S^- and S^+	Discrimination between S^- (Signal-ing water) and S^+ (signaling cat milk): touching a target within 10 sec with the snout to receive the US; Criterion: S^+ latency > S^-latency per dog (Mann-Whitney U-test).<br>15 of the 23 dogs successfully passed training	15 S^- and S^+ trials, and 2 times the 9 ambiguous probes were presented pseudo-randomly. No reward was given; test was repeated twice over the course of 2 weeks	✓		✓	Large breed differences in the latencies (and likelihood) to touch a target. Shorter latencies (and higher likelihood to touch target) for S^+ and probes near S^+ and longer latencies (and lower likelihood to touch target) for S^- and probes near S^-<br>Dogs appeared to learn that responding during testing was unrewarded.	(Starling, 2012)
Dogs, various breeds	f, m	Investigate baseline optimism in dogs from different environ-ments: companion dogs, dogs in training for assistance roles and security/detection dogs	1	16	a;<br>S^- high or low tone signaling water as reward<br>S^+ the other of the two tones, signaling lactose free milk as reward	a(9); tones between the ones associ-ated with S^- and S^+	Discrimination between S^- (signaling water) and S^+ (signaling cat milk): touching a target within 10 sec with the snout to receive the US; Criterion: S^+ latency > S^-latency per dog (Mann-Whitney U-test).<br>20 of the 40 dogs successfully passed training	15 S^- and S^+ trials, and 2 times the 9 ambiguous probes (tones in between S^+ and S^-) were presented pseudo-randomly. No reward was given; test was repeated 3 times over the course of 2 weeks	✓		✓	Dogs were slower to touch the target as probes became more similar to S^-. Inter-individual differences in responses to ambiguous probes, also between dogs from the same treatment group.	(Starling et al., 2014)
Cats, domestic shorthair	f, m [22]	Individual differences between cats.		F	s;<br>S^- bucket on one side of the arena);<br>S^+ bucket on the other side	s(3); buckets in between S^- and S^+ location	Discrimination between location of S^- (inaccessible food) and S^+ (buck-et containing the food reward); Criterion: statistically significant difference in latency to approach S^-, S^+ on two consecutive days	3 consecutive days, with 13 trials (5 rewarded, 5 unreward-ed, 3 unrewarded ambiguous locations, in between S^+ and S^-)		✓	✓	Strong differences between cats to discriminate between rewarded and unrewarded locations.<br>Shorter latencies for S^+ and probes near S^+ and longer latencies (and lower likelihood to touch target) for S^- and probes near S^-	(Tami et al., 2011)
Dogs, various breeds	f, m	Dogs kenneled for > 6 months 'long term' (LT) group, vs. dogs kenneled between approximate-ly 1 week and 3 months 'short term' (ST). Groups were matched for age, sex, breed and breeding status	1	F	s; S^- bucket on one side of the arena);<br>S^+ bucket on the other side	s(3); buckets in between S^- and S^+ location	Discrimination between location of S^- (empty bucket) and S^+ (bucket containing the food reward); Criterion: shorter latency to ap-proach the S^+ than the S- location in each trial of a series of 6	3 series of tests with ambigu-ous locations, followed by the originally trained S^- and S^+ locations twice, to re-establish the original discrimination		✓	✓	No effects of the LT and ST group on latencies to approach the different bucket locations.<br>Shorter latencies for S^+ and probes near S^+ and longer latencies (and lower likelihood to touch target) for S^- and probes near S^-	(Titulaer et al., 2013)

[21] Operant apparatus, equipped with a touch area and a milk/lactose delivery system

[22] All neutered

Species (strain/breed)	Sex	Experimental manipulation(s)	When	Test-arena	S^-/S^+	Ambiguous stimuli (nr.)	Training	Testing	Go/ No-go	Go/Go	Welfare	Effect(s) of experimental manipulation(s)	Reference
Sheep, Merino ewes	f	Group 1: sheep fasted for 24 h before cognitive bias testing; Group 2: sheep treated with ghrelin (7µg.kg^{-1} body weight); Group 3: untreated controls (Experiment 1)	2	D	v; S^- green panel of very high or low brightness, S^+ panel with brightness opposite to S^-,	v(3); green panels with brightness between S^- and S^+	Training to approach the S^+ location (, exposure to 2 sheep when the panel is raised) and to avoid the S- location (exposes the sheep to a dog when the panel is raised), where no-go was defined as not approaching the locations for 30 seconds.	Five consecutive trials with the five different cue locations: S^- and S^+ location were reinforced, ambiguous cue locations had no consequences	✓		✓	100% approaches to S^+ location, very low proportion of approaches to S^- location. Tendency to more pessimistic bias in ghrelin treated sheep.	(Verbeek et al., 2014a)
Sheep, Merino ewes,	f	Group 1: high feed (HF): increasing feed amount from 110 to 150% of required maintenance level (days 1-4), followed by supplying 170% (days 4-8). Group 2: low feed (LF): food deprivation (day 1), followed by supplying approx. 50% of maintenance level (day 2- 6). (Experiment 2)	2	D	v; S^- green panel of very high or low brightness, S^+ panel with brightness opposite to S^-,	v(3); green panels with brightness between S^- and S^+	Exp. 2 followed exp.1 within one week Training to approach the S^+ location (, exposure to 2 sheep when the panel is raised) and to avoid the S- location (exposes the sheep to a dog when the panel is raised), where no-go was defined as not approaching the locations for 30 seconds.	Five consecutive trials with the five different cue locations: S^- and S^+ location were reinforced, ambiguous cue locations had no consequences	✓		✓	The HF group tended to approach all locations less often than the LF group, i.e. LF sheep appeared to have a more optimistic judgement than the HF sheep. Group 1: Cognitive bias testing on day 7, 3 h after feeding. Group 2: Food deprivation on day 7, cognitive bias testing on day 7	(Verbeek et al., 2014a)
Sheep, Merino ewes	f	Group 1: morphine (1 mg.kg^{-1} body weight) i.v. Group 2: naloxone (2 mg.kg^{-1} body weight) i.v. Group 3: controls, receiving 10 ml sterile water i.v. Injections 10 minutes before the start of cognitive bias testing. Sheep received either palatable food pellets (70 grams) or unpalatable food (wood chips) in the start box, before they were released into the testing arena, counterbalanced for half of the animals on day 1 and 2 of testing.	2	D	v; S^- green panel of very high or low brightness, S^+ panel with brightness opposite to S^-,	v(3); green panels with brightness between S^- and S^+	Training to approach the S^+ location (green panel of very high or low brightness, exposure to 2 sheep when the panel is raised) and to avoid the S- location (other brightness that exposes the sheep to a dog when the panel is raised), where no-go was defined as not approaching the locations for 30 seconds.	Two testing days, separated by one day rest. Per testing day: five consecutive trials with the five different cue locations in random order: S^- and S^+ location was reinforced, approaching an ambiguous cue locations had no consequences	✓			Strong carry over effects of cognitive bias testing on day 2 of the testing on day 1. Day 2 data therefore were not analyzed. Ambiguous cues were approached sooner when animals were exposed to palatable food in the startbox and this effect seemed to be strengthened by morphine, however no differences in pessimism were found between the control group and morphine treated animals indicating that the wood chips were not less aversive for morphine treated animals. Also no differences in optimism were found between the naloxone and control group	(Verbeek et al., 2014b)

Species (strain/breed)	Sex	Experimental manipulation(s)	When	Test-arena	S^-/S^+	Ambiguous stimuli (nr.)	Training	Testing	Go/ No-go	Go/Go	Welfare	Effect(s) of experimental manipulation(s)	Reference
Sheep, Laucune ewes	f	Housing in either unpredictable, stimulus-poor environment or predictable, stimulus-rich environment for a duration of several months.	1	D	s; S^+/S^- boxes presented in different locations	s(3); boxes presented in intermediate positions between S^+/S^- locations	Approach box at S^+ (one side of the room, rewarded with food)/avoid approach at S^- (other side of the room, punished by presenting blower with cloth attached), training continued until a sheep performed 15 correct responses in a row (9 positive, 6 negative in semi-random order). Eleven of 12 sheep from predictable group and 7 of 12 sheep from unpredictable group reached criterion.	Three test days, test sequence of five trials containing 1 ambiguous stimulus presented between S^+/S^-, each day a different ambiguous location. ambiguous stimuli were unrewarded	✓		✓	Sheep from predictable group needed fewer training sessions than sheep from unpredictable group. Sheep from unpredictable group were less likely to approach middle and near-positive ambiguous stimulus but more likely to approach near-negative ambiguous stimulus	(Vögeli et al., 2014)
Dogs, various breeds	f, m[12]	Removal of conspecific in pair-housed dogs.	2	F	s; S^- bucket on one side of the arena); S^+ bucket on the other side	s(3); bowl in between S^- and S^+ location	Approach bowl at S^+ (bowl containing food reward)/refrain from approaching bowl at S^- (empty bowl). Training continued for a minimum of 15 trials in randomized order until the longest latency to reach S^+ was shorter than any of the 3 preceding latencies to reach S^-.	Testing prior to and after separation. During a test session 3 trials were undertaken for each ambiguous location (total of 9 ambiguous trials). Prior to and between ambiguous trials, 2 S^+ and 2 S^- trials were performed.	✓		✓	Latency to approach increased as bowl was placed nearer the S^- location. No effect of separation found on latencies to reach ambiguous bowl locations, indicating no change in emotional state.	(Walker et al., 2014)
Chickens *(gallus gallus)*	f	Housing in basic or enriched environment; housing in these environments started 3 days before testing (first subgroup) and 2 months before testing (second subgroup)	1	D	s; S^- bucket on one side of the arena); S^+ bucket on the other side	s(3); buckets in between S^- and S^+ location	Discrimination between location of S^- (empty bowl) and S^+ (bowl containing the food reward); Criterion: Latency to approach the S^- 5 s longer than approaching S^+ location (in at least 3 out of 4 times that the bowl was in the S^+ position, in the 8 trials of a training session)	3 days each separated by one test free day, with 13 trials (5 rewarded, 5 unrewarded, 3 unrewarded ambiguous locations		✓	✓	Training on the cognitive bias task was time consuming (approx. 150 trial needed to reach training criterion). The housing conditions did not affect proportion of chicks (with cut off 20 sec), nor the latencies to approach the different bowl locations.	(Wichman et al., 2012)

References

Anderson, M. H., Munafò, M. R., and Robinson, E. S. J. (2013). Investigating the psychopharmacology of cognitive affective bias in rats using an affective tone discrimination task. *Psychopharmacology (Berl.)* 226, 601–613. doi:10.1007/s00213-012-2932-5.

Ash, H., and Buchanan-Smith, H. M. (2016). The long-term impact of infant rearing background on the affective state of adult common marmosets (Callithrix jacchus). *Appl. Anim. Behav. Sci.* 174, 128–136. doi:10.1016/j.applanim.2015.10.009.

Barker, T. H., Howarth, G. S., and Whittaker, A. L. (in press). The effects of metabolic cage housing and sex on cognitive bias expression in rats. *Appl. Anim. Behav. Sci.* XX, xxx–xxx. doi:10.1016/j.applanim.2016.01.018.

Bateson, M., Desire, S., Gartside, S. E., and Wright, G. A. (2011). Agitated Honeybees Exhibit Pessimistic Cognitive Biases. *Curr. Biol.* 21, 1070–1073. doi:10.1016/j.cub.2011.05.017.

Bateson, M., Emmerson, M., Ergün, G., Monaghan, P., and Nettle, D. (2015). Opposite effects of early-life competition and developmental telomere attrition on cognitive biases in juvenile European starlings. *PLoS ONE* 10, e0132602. doi:10.1371/journal.pone.0132602.

Bateson, M., and Matheson, S. M. (2007). Performance on a categorisation task suggests that removal of environmental enrichment induces “pessimism” in captive European starlings (Sturnus vulgaris). *Anim. Welf.* 16, 33–36.

Bateson, M., and Nettle, D. (2015). Development of a cognitive bias methodology for measuring low mood in chimpanzees. *PeerJ Prepr.* 3, 25. doi:10.7717/peerj.998.

Bethell, E. J., Holmes, A., MacLarnon, A., and Semple, S. (2012). Cognitive bias in a non-human primate: husbandry procedures influence cognitive indicators of psychological well-being in captive rhesus macaques. *Anim. Welf.* 21, 185–195. doi:10.7120/09627286.21.2.185.

Bethell, E. J., and Koyama, N. F. (2015). Happy hamsters? Enrichment induces positive judgement bias for mildly (but not truly) ambiguous cues to reward and punishment in Mesocricetus auratus. *R. Soc. Open Sci.* 2, 140399. doi:10.1098/rsos.140399.

Boleij, H. (2013). Emotional perceptions in mice: studies on judgement bias and behavioural habituation. Chapter 6: Food avoidance in mice: effects of quinine and denatonium benzoate. Available at: http://dspace.library.uu.nl/handle/1874/276185.

Boleij, H., van ’t Klooster, J., Lavrijsen, M., Kirchhoff, S., Arndt, S. S., and Ohl, F. (2012). A test to identify judgement bias in mice. *Behav. Brain Res.* 233, 45–54. doi:10.1016/j.bbr.2012.04.039.

Brajon, S., Laforest, J.-P., Schmitt, O., and Devillers, N. (2015). The way humans behave medulates the Emotional state of piglets. *PloS ONE* 10, e0133408. doi:10.1371/journal.pone.0133408.

Briefer, E. F., and McElligott, A. G. (2013). Rescued goats at a sanctuary display positive mood after former neglect. *Appl. Anim. Behav. Sci.* 146, 45–55. doi:10.1016/j.applanim.2013.03.007.

Briefer Freymond, S., Briefer, E. F., Zollinger, A., Gindrat-von Allmen, Y., Wyss, C., and Bachmann, I. (2014). Behaviour of horses in a judgment bias test associated with positive or negative reinforcement. *Appl. Anim. Behav. Sci.* 158, 34–45. doi:10.1016/j.applanim.2014.06.006.

Brilot, B. O., Asher, L., and Bateson, M. (2010). Stereotyping starlings are more “pessimistic.” *Anim. Cogn.* 13, 721–731. doi:10.1007/s10071-010-0323-z.

Brilot, B. O., Normandale, C. L., Parkin, A., and Bateson, M. (2009). Can we use starlings’ aversion to eyespots as the basis for a novel “cognitive bias” task? *Appl. Anim. Behav. Sci.* 118, 182–190. doi:10.1016/j.applanim.2009.02.015.

Brydges, N. M., Hall, L., Nicolson, R., Holmes, M. C., and Hall, J. (2012). The effects of juvenile stress on anxiety, cognitive bias and decision making in adulthood: a rat model. *PLoS ONE* 7, e48143. doi:10.1371/journal.pone.0048143.

Brydges, N. M., Leach, M., Nicol, K., Wright, R., and Bateson, M. (2011). Environmental enrichment induces optimistic cognitive bias in rats. *Anim. Behav.* 81, 169–175. doi:10.1016/j.anbehav.2010.09.030.

Burman, O. H. P., Parker, R. M. A., Paul, E. S., and Mendl, M. T. (2009). Anxiety-induced cognitive bias in non-human animals. *Physiol. Behav.* 98, 345–350. doi:10.1016/j.physbeh.2009.06.012.

Burman, O. H. P., Parker, R., Paul, E. S., and Mendl, M. (2008). A spatial judgement task to determine background emotional state in laboratory rats, rattus norvegicus. *Anim. Behav.* 76, 801–809. doi:10.1016/j.anbehav.2008.02.014.

Burman, O., McGowan, R., Mendl, M., Norling, Y., Paul, E., Rehn, T., et al. (2011). Using judgement bias to measure positive affective state in dogs. *Appl. Anim. Behav. Sci.* 132, 160–168. doi:10.1016/j.applanim.2011.04.001.

Carreras, R., Arroyo, L., Mainau, E., Peña, R., Bassols, A., Dalmau, A., et al. (in press). Effect of gender and halothane genotype on cognitive bias and its relationship with fear in pigs. *Appl. Anim. Behav. Sci.* XX, xxx–xxx. doi:10.1016/j.applanim.2016.01.019.

Carreras, R., Mainau, E., Rodriguez, P., Llonch, P., Dalmau, A., Manteca, X., et al. (2015). Cognitive bias in pigs: individual classification and consistency over time. *J. Vet. Behav.* 10, 577–581. doi:10.1016/j.jveb.2015.09.001.

Coulon, M., Nowak, R., Andanson, S., Petit, B., Lévy, F., and Boissy, A. (2015). Effects of prenatal stress and emotional reactivity of the mother on emotional and cognitive abilities in lambs. *Dev. Psychobiol.* 57, 626–636. doi:10.1002/dev.21320.

Daros, R. R., Costa, J. H. C., von Keyserlingk, M. A. G., Hötzel, M. J., and Weary, D. M. (2014). Separation from the dam causes negative judgement bias in dairy calves. *PLoS ONE* 9, e98429. doi:10.1371/journal.pone.0098429.

Destrez, A., Deiss, V., Belzung, C., Lee, C., and Boissy, A. (2012). Does reduction of fearfulness tend to reduce pessimistic-like judgment in lambs? *Appl. Anim. Behav. Sci.* 139, 233–241. doi:10.1016/j.applanim.2012.04.006.

Destrez, A., Deiss, V., Leterrier, C., Calandreau, L., and Boissy, A. (2014). Repeated exposure to positive events induces optimistic-like judgment and enhances fearfulness in chronically stressed sheep. *Appl. Anim. Behav. Sci.* 154, 30–38. doi:10.1016/j.applanim.2014.01.005.

Destrez, A., Deiss, V., Lévy, F., Calandreau, L., Lee, C., Chaillou-Sagon, E., et al. (2013). Chronic stress induces pessimistic-like judgment and learning deficits in sheep. *Appl. Anim. Behav. Sci.* 148, 28–36. doi:10.1016/j.applanim.2013.07.016.

Douglas, C., Bateson, M., Walsh, C., Bédué, A., and Edwards, S. A. (2012). Environmental enrichment induces optimistic cognitive biases in pigs. *Appl. Anim. Behav. Sci.* 139, 65–73. doi:10.1016/j.applanim.2012.02.018.

Doyle, R. E., Fisher, A. D., Hinch, G. N., Boissy, A., and Lee, C. (2010a). Release from restraint generates a positive judgement bias in sheep. *Appl. Anim. Behav. Sci.* 122, 28–34. doi:10.1016/j.applanim.2009.11.003.

Doyle, R. E., Hinch, G. N., Fisher, A. D., Boissy, A., Henshall, J. M., and Lee, C. (2011a). Administration of serotonin inhibitor p-Chlorophenylalanine induces pessimistic-like judgement bias in sheep. *Psychoneuroendocrinology* 36, 279–288. doi:10.1016/j.psyneuen.2010.07.018.

Doyle, R. E., Lee, C., Deiss, V., Fisher, A. D., Hinch, G. N., and Boissy, A. (2011b). Measuring judgement bias and emotional reactivity in sheep following long-term exposure to unpredictable and aversive events. *Physiol. Behav.* 102, 503–510. doi:10.1016/j.physbeh.2011.01.001.

Doyle, R. E., Vidal, S., Hinch, G. N., Fisher, A. D., Boissy, A., and Lee, C. (2010b). The effect of repeated testing on judgement biases in sheep. *Behav. Processes* 83, 349–352. doi:10.1016/j.beproc.2010.01.019.

Düpjan, S., Ramp, C., Kanitz, E., Tuchscherer, A., and Puppe, B. (2013). A design for studies on cognitive bias in the domestic pig. *J. Vet. Behav. Clin. Appl. Res.* 8, 485–489. doi:10.1016/j.jveb.2013.05.007.

Enkel, T., Gholizadeh, D., von Bohlen und Halbach, O., Sanchis-Segura, C., Hurlemann, R., Spanagel, R., et al. (2010). Ambiguous-cue interpretation is biased under stress- and depression-like states in rats. *Neuropsychopharmacology* 35, 1008–1015. doi:10.1038/npp.2009.204.

Fernandes, J. M. G. M. (2012). Developing visual discrimination tasks for dogs. Available at: http://repositorio.ul.pt/bitstream/10451/7368/1/ulfc099186_tm_joana_fernandes.pdf.

Gordon, D. J., and Rogers, L. J. (2015). Cognitive bias, hand preference and welfare of common marmosets. *Behav. Brain Res.* 287, 100–108. doi:10.1016/j.bbr.2015.03.037.

Guldimann, K., Vögeli, S., Wolf, M., Wechsler, B., and Gygax, L. (2015). Frontal brain deactivation during a non-verbal cognitive judgement bias test in sheep. *Brain Cogn.* 93, 35–41. doi:10.1016/j.bandc.2014.11.004.

Harding, E. J., Paul, E. S., and Mendl, M. (2004). Animal behaviour — cognitive bias and affective state. *Nature* 427, 312. doi:10.1038/427312a.

Hernandez, C. E., Hinch, G., Lea, J., Ferguson, D., and Lee, C. (2015). Acute stress enhances sensitivity to a highly attractive food reward without affecting judgment bias in laying hens. *Appl. Anim. Behav. Sci.* 163, 135–143. doi:10.1016/j.applanim.2014.12.002.

Hymel, K. A., and Sufka, K. J. (2012). Pharmacological reversal of cognitive bias in the chick anxiety-depression model. *Neuropharmacology* 62, 161–166. doi:10.1016/j.neuropharm.2011.06.009.

Karagiannis, C. I., Burman, O. H. P., and Mills, D. S. (2015). Dogs with separation-related problems show a "less pessimistic" cognitive bias during treatment with fluoxetine (Reconcile^{TM}) and a behaviour modification plan. *BMC Vet. Res.* 11, 10. doi:10.1186/s12917-015-0373-1.

Keen, H. A., Nelson, O. L., Robbins, C. T., Evans, M., Shepherdson, D. J., and Newberry, R. C. (2014). Validation of a novel cognitive bias task based on difference in quantity of reinforcement for assessing environmental enrichment. *Anim. Cogn.* 17, 529–541. doi:10.1007/s10071-013-0684-1.

Kis, A., Hernádi, A., Kanizsár, O., Gácsi, M., and Topál, J. (2015). Oxytocin induces positive expectations about ambivalent stimuli (cognitive bias) in dogs. *Horm. Behav.* 69, 1–7. doi:10.1016/j.yhbeh.2014.12.004.

Kloke, V., Schreiber, R. S., Bodden, C., Möllers, J., Ruhmann, H., Kaiser, S., et al. (2014). Hope for the best or prepare for the worst? towards a spatial cognitive bias test for mice. *PLoS ONE* 9, e105431. doi:10.1371/journal.pone.0105431.

Kregiel, J., Golebiowska, J., Popik, P., and Rygula, R. (2016a). Dopamine induces an optimism bias in rats – pharmacological proof for the translational validity of the ambiguous-cue interpretation test. *Behav. Brain Res.* 297, 84–90. doi:10.1016/j.bbr.2015.10.020.

Kregiel, J., Malek, N., Popik, P., Starowicz, K., and Rygula, R. (2016b). Anandamide mediates cognitive judgement bias in rats. *Neuropharmacology* 101, 146–153. doi:10.1016/j.neuropharm.2015.09.009.

Lindström, L. (2010). Performance of laying hens in a cognitive bias task; the effect of time since change of environment. Available at: http://stud.epsilon.slu.se/863/3/lindstrom_l_100312.pdf.

Matheson, S. M., Asher, L., and Bateson, M. (2008). Larger, enriched cages are associated with "optimistic" response biases in captive European starlings (Sturnus vulgaris). *Appl. Anim. Behav. Sci.* 109, 374–383. doi:10.1016/j.applanim.2007.03.007.

McGuire, M. C., Williams, K. L., Welling, L. L. M., and Vonk, J. (2015). Cognitive bias in rats is not influenced by oxytocin. *Front. Psychol.* 6, 12. doi:10.3389/fpsyg.2015.01306.

Mendl, M., Brooks, J., Basse, C., Burman, O., Paul, E., Blackwell, E., et al. (2010). Dogs showing separation-related behaviour exhibit a "pessimistic" cognitive bias. *Curr. Biol.* 20, R839–R840. doi:10.1016/j.cub.2010.08.030.

Müller, C. A., Riemer, S., Rosam, C. M., Schößwender, J., Range, F., and Huber, L. (2012). Brief owner absence does not induce negative judgement bias in pet dogs. *Anim. Cogn.* 15, 1031–1035. doi:10.1007/s10071-012-0526-6.

Murphy, E., Nordquist, R. E., and van der Staay, F. J. (2013). Responses of conventional pigs and Göttingen miniature pigs in an active choice judgement bias task. *Appl. Anim. Behav. Sci.* 148, 64–76. doi:10.1016/j.applanim.2013.07.011.

Neave, H. W., Daros, R. R., Costa, J. H. C., von Keyserlingk, M. A. G., and Weary, D. M. (2013). Pain and pessimism: dairy calves exhibit negative judgement bias following hot-iron disbudding. *PLoS ONE* 8, e80556. doi:10.1371/journal.pone.0080556.

Nogueira, S. S. da C., Fernandes, I. K., Costa, T. S. O., Nogueira-Filho, S. L. G., and Mendl, M. (2015). Does trapping influence decision-making under ambiguity in white-lipped peccary (Tayassu pecari)? *PloS ONE* 10, e0127868. doi:10.1371/journal.pone.0127868.

Novak, J., Bailoo, J. D., Melotti, L., Rommen, J., and Würbel, H. (2015). An exploration based cognitive bias test for mice: effects of handling method and stereotypic behaviour. *PLoS ONE* 10, e0130718. doi:10.1371/journal.pone.0130718.

Novak, J., Stojanovski, K., Melotti, L., Reichlin, T. S., Palme, R., and Würbel, H. (2016). Effects of stereotypic behaviour and chronic mild stress on judgement bias in laboratory mice. *Appl. Anim. Behav. Sci.* 174, 162–172. doi:10.1016/j.applanim.2015.10.004.

Papciak, J., Popik, P., Fuchs, E., and Rygula, R. (2013). Chronic psychosocial stress makes rats more "pessimistic" in the ambiguous-cue interpretation paradigm. *Behav. Brain Res.* 256, 305–310. doi:10.1016/j.bbr.2013.08.036.

Parker, R. M. A., Paul, E. S., Burman, O. H. P., Browne, W. J., and Mendl, M. (2014). Housing conditions affect rat responses to two types of ambiguity in areward–reward discrimination cognitive bias task. *Behav. Brain Res.* 274, 73–83. doi:10.1016/j.bbr.2014.07.048.

Pomerantz, O., Terkel, J., Suomi, S. J., and Paukner, A. (2012). Stereotypic head twirls, but not pacing, are related to a "pessimistic"-like judgment bias among captive tufted capuchins (Cebus apella). *Anim. Cogn.* 15, 689–698. doi:10.1007/s10071-012-0497-7.

Rafa, D., Kregiel, J., Popik, P., and Rygula, R. (2016). Effects of optimism on gambling in the rat slot machine task. *Behav. Brain Res.* 300, 97–105.

Richter, S. H., Schick, A., Hoyer, C., Lankisch, K., Gass, P., and Vollmayr, B. (2012). A glass full of optimism: Enrichment effects on cognitive bias in a rat model of depression. *Cogn. Affect. Behav. Neurosci.* 12, 527–542. doi:10.3758/s13415-012-0101-2.

Rygula, R., Golebiowska, J., Kregiel, J., Holuj, M., and Popik, P. (2015a). Acute administration of lithium, but not valproate, modulates cognitive judgment bias in rats. *Psychopharmacology (Berl.)* 232, 2149–2156. doi:10.1007/s00213-014-3847-0.

Rygula, R., Kregiel, J., Golebiowska, J., Kubik, J., and Popik, P. (2015b). Effects of optimism on motivation in rats. *Front. Behav. Neurosci.* 9. doi:10.3389/fnbeh.2015.00032.

Rygula, R., Papciak, J., and Popik, P. (2013). Trait pessimism predicts vulnerability to stress-induced anhedonia in rats. *Neuropsychopharmacology* 38, 2188–2196. doi:10.1038/npp.2013.116.

Rygula, R., Papciak, J., and Popik, P. (2014a). The effects of acute pharmacological stimulation of the 5-HT, NA and DA systems on the cognitive judgement bias of rats in the ambiguous-cue interpretation paradigm. *Eur. Neuropsychopharmacol.* 24, 1103–1111. doi:10.1016/j.euroneuro.2014.01.012.

Rygula, R., Pluta, H., and Popik, P. (2012). Laughing rats are optimistic. *PLoS ONE* 7, e51959. doi:10.1371/journal.pone.0051959.

Rygula, R., Szczech, E., Kregiel, J., Golebiowska, J., Kubik, J., and Popik, P. (2015c). Cognitive judgement bias in the psychostimulant-induced model of mania in rats. *Psychopharmacology (Berl.)* 232, 651–660. doi:10.1007/s00213-014-3707-y.

Rygula, R., Szczech, E., Papciak, J., Nikiforuk, A., and Popik, P. (2014b). The effects of cocaine and mazindol on the cognitive judgement biasof rats in the ambiguous-cue interpretation paradigm. *Behav. Brain Res.* 270, 206–212. doi:10.1016/j.bbr.2014.05.026.

Salmeto, A. L., Hymel, K. A., Carpenter, E. C., Brilot, B. O., Bateson, M., and Sufka, K. J. (2011). Cognitive bias in the chick anxiety–depression model. *Brain Res.* 1373, 124–130. doi:10.1016/j.brainres.2010.12.007.

Sanger, E., Doyle, R. E., Hinch, G. N., and Lee, C. (2011). Sheep exhibit a positive judgement bias and stress-induced hyperthermia following shearing. *Appl. Anim. Behav. Sci.* 131, 94–103. doi:10.1016/j.applanim.2011.02.001.

Scollo, A., Gottardo, F., Contiero, B., and Edwards, S. A. (2014). Does stocking density modify affective state in pigs as assessed by cognitive bias, behavioural and physiological parameters? *Appl. Anim. Behav. Sci.* 153, 26–35. doi:10.1016/j.applanim.2014.01.006.

Seehuus, B., Mendl, M., Keeling, L. J., and Blokhuis, H. (2013). Disrupting motivational sequences in chicks: are there affective consequences? *Appl. Anim. Behav. Sci.* 148, 85–92. doi:10.1016/j.applanim.2013.07.008.

Starling, M. (2012). A portable, automated apparatus for testing cognitive bias in dogs. in *Proceedings of the AIAM Annual Conference on Animal Management, 2012* (Penrith), 40–46.

Starling, M. J., Branson, N., Cody, D., Starling, T. R., and McGreevy, P. D. (2014). Canine sense and sensibility: tipping points and response latency variability as an optimism index in a canine judgement bias sssessment. *PLoS ONE* 9, e107794. doi:10.1371/journal.pone.0107794.

Tami, G., Torre, C., Compagnucci, M., and Manteca, X. (2011). Interpretation of ambiguous spatial stimuli in cats. *Anim. Welf.* 20, 185–189.

Titulaer, M., Blackwell, E. J., Mendl, M., and Casey, R. A. (2013). Cross sectional study comparing behavioural, cognitive and physiological indicators of welfare between short and long term kennelled domestic dogs. *Appl. Anim. Behav. Sci.* 147, 149–158. doi:10.1016/j.applanim.2013.05.001.

Verbeek, E., Ferguson, D., and Lee, C. (2014a). Are hungry sheep more pessimistic? The effects of food restriction on cognitive bias and the involvement of ghrelin in its regulation. *Physiol. Behav.* 123, 67–75. doi:10.1016/j.physbeh.2013.09.017.

Verbeek, E., Ferguson, D., Quinquet de Monjour, P., and Lee, C. (2014b). Generating positive affective states in sheep: the influence of food rewards and opioid administration. *Appl. Anim. Behav. Sci.* 154, 39–47. doi:10.1016/j.applanim.2014.02.011.

Vögeli, S., Lutz, J., Wolf, M., Wechsler, B., and Gygax, L. (2014). Valence of physical stimuli, not housing conditions, affects behaviour and frontal cortical brain activity in sheep. *Behav. Brain Res.* 267, 144–155. doi:10.1016/j.bbr.2014.03.036.

Walker, J. K., Waran, N. K., and Phillips, C. J. C. (2014). The effect of conspecific removal on the behaviour and physiology of pair-housed shelter dogs. *Appl. Anim. Behav. Sci.* 158, 46–56. doi:10.1016/j.applanim.2014.06.010.

Wichman, A., Keeling, L. J., and Forkman, B. (2012). Cognitive bias and anticipatory behaviour of laying hens housed in basic and enriched pens. *Appl. Anim. Behav. Sci.* 140, 62–69. doi:10.1016/j.applanim.2012.05.006.
